# Assignment of Canadian Defined Daily Doses and Canadian Defined Course Doses for Quantification of Antimicrobial Usage in Cattle

**DOI:** 10.3389/fvets.2020.00010

**Published:** 2020-01-29

**Authors:** Hélène Lardé, Simon Dufour, Marie Archambault, David Léger, Daleen Loest, Jean-Philippe Roy, David Francoz

**Affiliations:** ^1^Department of Pathology and Microbiology, Faculty of Veterinary Medicine, Université de Montréal, Saint-Hyacinthe, QC, Canada; ^2^Fonds de Recherche du Québec – Nature et Technologies Strategic Cluster Op+lait, Regroupement de Recherche Pour un Lait de Qualité Optimale, Saint-Hyacinthe, QC, Canada; ^3^Centre for Food-borne, Environmental and Zoonotic Infectious Diseases, Public Health Agency of Canada, Guelph, ON, Canada; ^4^Department of Clinical Sciences, Faculty of Veterinary Medicine, Université de Montréal, Saint-Hyacinthe, QC, Canada

**Keywords:** antimicrobial usage, antibiotic usage, animal infection, cattle, metrics, DDDbovCA, DCDbovCA, quantification

## Abstract

Standardized units are essential to allow quantification and comparison of antimicrobial usage (AMU) between species and regions. In Canada, defined daily and course doses have not yet been harmonized for cattle. Our objective was to assign defined daily and course doses (named DDDbovCA and DCDbovCA, respectively) for cattle in Canada, by antimicrobial agent (AM) and by route of administration, based on the label of all products containing at least one AM, marketed and authorized in Canada for use in cattle. In April and December 2019, a systematic search was performed from the online Drug Product Database (DPD) of Health Canada to identify veterinary products containing at least one AM, marketed in Canada for use in cattle. Products were divided by route of administration (intramammary, intrauterine, injectable, oral, and topical). The monograph was retrieved for each product from the DPD, or from the Canadian Edition of the Compendium of Veterinary Products (CVP), and read completely to extract recommended dosages in cattle. Standard weights were applied to compute doses if required. DDDbovCA and DCDbovCA were assigned by calculating an average of daily and course doses, respectively, by AM and route of administration. Two products were excluded from calculations because of their claim as growth promotion or feed efficiency (no longer authorized in Canada for certain categories of AM). Overall, 39 injectable, 75 oral (including 23 medicated premixes), 8 intramammary (4 for lactating cows and 4 for dry cows), 5 intrauterine, and 4 topical products were used for calculations. DDDbovCA and DCDbovCA values were assigned successfully for each AM identified, by route of administration. These metrics will allow harmonized and transparent quantification of AMU in cattle in Canada.

## Introduction

With increasing interest in evaluation of the impact of antimicrobial usage (AMU) on antimicrobial resistance, international health organizations have highlighted the importance to monitor AMU in human and veterinary medicine, as well as in agriculture (1–3). Since, the early 2000s, countries have reported their AMU for animals (4–8). At the same time, problems of comparability between methods of quantification and between units of measurement were raised (9–12). Nowadays, standardization of indicators is targeted by public health authorities (13–15).

Quantities of antimicrobial agents (AMs) used can be reported in net mass or in number of standard doses per standardized biomass (16) or per animal or group of animals (17). To account for differences in potency and molecular weight between different AMs, standard doses are often preferred over net masses to report quantities. Different standard doses have been proposed: defined doses (18), used (or actual) doses (19), and prescribed doses (20). Cow Calculated Course is a recent metric conceived in the United Kingdom that stratifies AMU for young cattle (long-acting injectable and oral products) and adult cattle (intramammary and short-acting injectable products) by assuming certain products are only used in certain age groups (21). The use of one standard dose instead of another depends on the source of data collection and the aim of the report on AMU.

In this context, the European Medicines Agency (EMA), through the European Surveillance of Veterinary Antimicrobial Consumption (ESVAC) project, assigned defined daily and course doses for animals (DDDvet and DCDvet), by food-producing species (cattle, swine, poultry), route of administration (parenteral, oral, intramammary for lactating cows, intramammary for dry cows, intrauterine), and AM or combination of AMs (22). They followed principles already established by the World Health Organization (WHO) for assignment of Defined Daily Doses (DDD) for human medicines (23). Canadian defined daily doses for animals (DDDvetCAs) have recently been defined for poultry (broiler chickens and turkeys) and pigs (24). Defined doses have also been used for reporting on Canadian AMU in dairy cattle (25, 26) and in beef cattle (27), but are not harmonized between authors.

The objective of this research was, therefore, to assign defined daily doses (named DDDbovCA) and defined course doses (named DCDbovCA) for cattle in Canada, based on the labeled doses of all products containing at least one AM, that are marketed and authorized for use in cattle in Canada. Specifically, the aim of this work was to assign DDDbovCA and DCDbovCA values by AM and by route of administration, in order to quantify in a transparent way AMU in cattle in Canada.

## Materials and Methods

### Database Search and Classification of Products by Route of Administration

**Figure 1:**
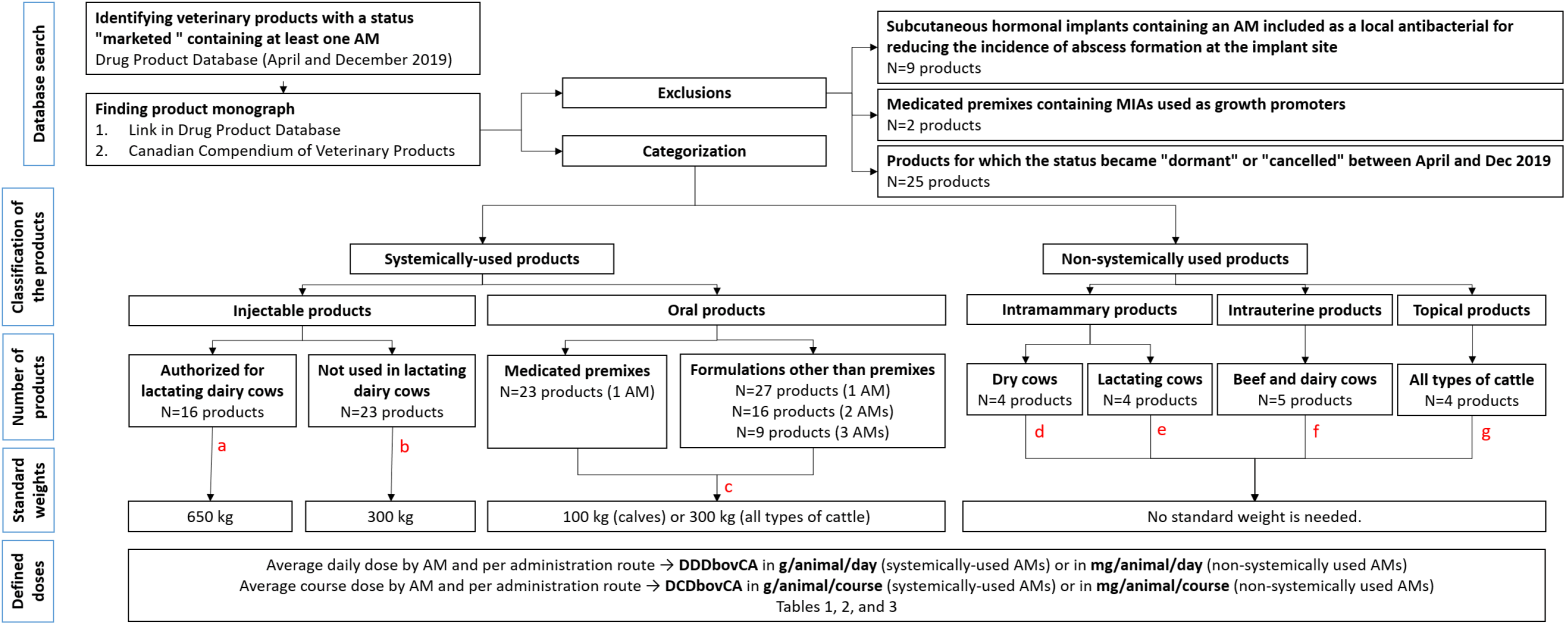
Flow chart illustrating workflow for proposing DDDbovCA and DCDbovCA values for antimicrobial agents used for cattle in Canada. AM(s), Antimicrobial Agent(s); DDDbovCA, Canadian Defined Daily Dose for cattle; DCDbovCA, Canadian Defined Course Dose for cattle; MIA(s), Medically Important Antimicrobial(s) belonging to the Categories I, II, and III according to Health Canada. ^a^Injectable products authorized for lactating dairy cows were assigned to a standard weight of 650 kg. ^b^Injectable products not used in lactating dairy cows were assigned to a standard weight of 300 kg. ^c^Oral products intended for “calves” or “calves up to 136 kg” were assigned to a standard weight of 100 kg. Oral products intended for all types of cattle or for “calves up to 360 kg” were assigned to a standard weight of 300 kg. ^d^For intramammary products for dry cows, a complete treatment for a cow was defined as the infusion of four syringes (one per quarter) at drying off regardless of the product. The complete treatment (4 syringes) was assigned to a duration of action of 10 days meaning that the daily treatment was defined as the infusion of 0.4 syringe per cow, or 0.1 syringe per quarter. ^e^For intramammary products for lactating cows, it was hypothesized that one quarter is infected (and then treated) at a time by cow. ^f^For intrauterine products, when no indication could be found on the product monograph, a duration of action of 24 h was assigned by default. ^g^For topical products, we assumed that 1 mL is sprayed per second (for sprays), 5 g of cream is used per application (for creams), or 5 g of powder is used per application (for powders for topical administration).

The complete workflow for proposing DDDs and defined course doses is described in Figure 1. Health Canada, the federal institution responsible for regulating drugs to support public safety in Canada, provides an online Drug Product Database (DPD) updated nightly. Products defined as a drug under the Food and Drugs Act are identified by a unique Drug Identification Number (DIN), a computer-generated eight-digit number assigned by Health Canada to a drug product prior to being marketed (28). In April 2019, the DPD was searched by active antimicrobial ingredient to retrieve all products used for cattle containing at least one AM. The search was repeated in December 2019 to note any discrepancies. Only products with a status “marketed” were kept for further steps (this status refers to an active DIN that is currently sold in Canada). Then, for each product, the product monograph was consulted by following the link in the DPD. For products with no monograph available in the DPD, the Canadian Edition of the Compendium of Veterinary Products (CVP) was consulted (29). Products were separated according to their route of administration: systemic (oral or injectable) or non-systemic (intramammary, intrauterine, topical, or ophthalmic). Oral products were also classified according to their pharmaceutical form because of their diversity: “boluses, capsules, or tablets,” “suspensions or solutions,” “water soluble powders,” and “medicated premixes.” Subcutaneous hormonal implants containing an AM included as a local antibacterial for reducing the incidence of abscess formation at the implant site were excluded. Products containing an AM belonging to the Categories I, II, or III according to Health Canada (30), and having only growth promotion or feed efficiency indications were also excluded, as they are no longer marketed in Canada with this claim, since December 2018 (31).

### General Rules Applied to Each Product for Extraction of Dosages and Doses From the Product Monograph

The terms “dose” and “dosage” are often used interchangeably. For the current work, though, we used the following definitions: a dosage corresponds to the amount of active substance applied per kilogram of body weight, whereas a dose corresponds to the amount of an active substance administered to a single animal (13). Daily dosages and course dosages were defined for systemically-used AMs, and were expressed in milligrams per kilogram per day and in milligrams per kilogram per course of treatment, respectively. All dosages were rounded to one (dosages > 1 mg/kg) or two (dosages between 0 and 1 mg/kg) decimal place(s). Daily doses and course doses were expressed for systemically-used AMs in grams per animal per day and in grams per animal per course of treatment, respectively, rounded to two decimal places. Daily doses and course doses were expressed for non-systemically used AMs in milligrams per animal per day and in milligrams per animal per course of treatment, respectively, rounded to a whole number.

A combination of AMs in one product was analyzed as if each AM was found individually in different products. Exceptions were applied if the following three criteria were concomitantly encountered: the combination is always synergistic at the specific given ratio found in veterinary products; AND the combination is known to decrease the risk of antimicrobial resistance in comparison with the use of the individual AM; AND the AMs in the combination are never found alone in products marketed for cattle in Canada.

For long-acting products (i.e., products with duration of action or a duration between two administrations longer than 24 h), the daily dose was determined by dividing the amount of AM in one administration by the number of days between two administrations (for products with repeated administrations) or by the duration of action (in days) specified in the product monograph (for products with a single administration).

When both preventive and treatment dosages were indicated on the label, only the treatment dosage was used.

Conversion factors of 0.00012 and 0.00060 were applied to convert international units to milligrams for polymyxin B and penicillin G, respectively (32, 33). If a prodrug concentration was given in the product monograph, the prodrug was not converted into drug for calculations of dosages or doses, and was reported as such in tables.

### Rules Specific to Products Used Systemically (Injectable or Oral Products)

For each product, a daily dosage and a course dosage were obtained from the monograph by AM, in milligrams of AM per kilogram of body weight per day and per course, respectively. To convert dosage to dose, the dosage was multiplied by a standard weight. Two standard weights were used for injectable products: 300 kg for products not authorized for lactating dairy cows, and 650 kg for products authorized for lactating cows. Two standard weights were used for oral products: 100 kg for products intended for “calves” or “calves up to 136 kg,” and 300 kg for products intended for all types of cattle or for “calves up to 360 kg.” The 650-kg weight for an adult cow was decided according to recent data recording the weight of mature cows in Canada (34). The 100- and 300-kg weights for a calf up to 136 kg and for “a lambda cattle,” respectively, were the same weights used previously by Jensen et al. (18). The 100-kg weight represents the average weight between a newborn calf (around 50 kg) and a weaned calf (around 100 and 200 kg for a dairy and a beef calf, respectively). The 300-kg weight represents the average weight between a newborn calf and an adult cattle. It is also assumed to be representative of the average weight of a beef cattle entering a feedlot (200–300 kg for a feeder calf, and around 400 kg for a yearling).

For products with both a single-dose therapy and a multiple-dose therapy (danofloxacin, enrofloxacin, florfenicol), the course dose was determined by performing an average between both provided therapies. The daily dose was determined from the multiple-dose therapy only (duration of action easier to assess with repeated regimen).

For products with only a single-dose therapy, the course dose was equal to the dose provided. The daily dose was determined for beta-lactams by dividing the course dose (in g/animal/course) by the time (in number of days) the plasmatic concentration of the AM exceeds the Minimum Inhibitory Concentration (MIC) for pathogens targeted by the label (information read from the product monograph), and for tetracyclines by dividing the course dose (in g/animal/course) by the time (in number of days) of sustained antibiotic blood level action. For macrolides and fluoroquinolones (from products with only a single-dose therapy), the daily dose was determined by dividing the course dose by an arbitrary duration of 7 days based on the most likely duration of action for treatment of bovine respiratory diseases (35).

For oral products, only the individual treatment was used when both individual and group treatments were present on the label, because doses were more accurately determined from the individual treatment (less approximations used for calculations). When a loading dose was indicated, followed by several days of treatment at a maintenance dose, a course dose was first calculated, then divided by the number of days of treatment to obtain the daily dose. A daily water intake of 10% of the body weight was used if the dosage was given in quantity of medicated water provided daily to the animal [same approximation used by the ESVAC project, Appendix 4 in European Medicines Agency, European Surveillance of Veterinary Antimicrobial Consumption (36)]. For medicated premixes, arbitrary decisions had to be made to determine daily and course doses: the period of exposition to the medicated feed was fixed to 3 months when no other information was provided on the label. A rounded month of 30 days was used. A standardized animal consuming daily 2% of its body weight (on a 100% dry matter basis) was used to provide estimates of dosages, if required [same approximation used by the ESVAC project, Appendix 4 in European Medicines Agency, European Surveillance of Veterinary Antimicrobial Consumption (36)].

### Rules Specific to Products Used Non-systemically (Intramammary, Intrauterine, and Topical or Ophthalmic Products)

For each product, a daily dose and a course dose were obtained from the monograph by AM, in milligrams of AM per animal per day and per course, respectively. Some arbitrary decisions were made in order to assign a daily dose for every product. For intramammary products designed for lactating cows, it was hypothesized that one quarter at a time is infected (and thus treated) per animal. For intramammary products given at dry-off, the duration of action was set at 10 days. The actual duration of action was difficult to identify. Some data were available on persistence of antibiotic residues in milk: 8–21 days for cloxacillin benzathine (37, 38), 14–28 days for cephapirin benzathine (39), and 9 days for benzylpenicillin procaine (38), but no information was available on the time the antibiotics stay effective after drying-off at levels equal or higher than the MIC against the pathogens involved. Furthermore, the persistence of an antibiotic in the udder is affected by factors inherent to the product (such as the solubility of the antibiotic salt, the quantity of antibiotic infused, and the base in which it is formulated) (37) and, likely, by factors inherent to the cow (such as production at the time of drying-off, leaking milk after drying-off, chronic intramammary infection).

For intrauterine products, a duration of action of 24 h was assigned if no information was retrieved from the monograph. Finally, a duration of treatment of 5 days was hypothesized for topical products. These decisions were arbitrary made (but in agreement with the very scarce literature on this topic) in order to avoid missing values in assignment of defined doses. For topical products, it was assumed that 1 mL is sprayed per second as proposed by Postma et al. (40), and that 5 g of powder or of cream are applied on a wound per treatment.

### Assignment of Defined Daily and Course Doses

When different products containing the same AM had different labeled doses, an average dose of the unique doses was calculated by route of administration. Antimicrobial agents from combinations were assigned different values than AMs found alone in products because the dose of a given AM is often lower when combined in a product than in a product where it is found alone. For oral AMs, a distinction was made between AMs originating from medicated premixes and AMs originating from other pharmaceutical forms. For oral AMs, an average dose was first calculated by type of formulation (an average for boluses, for suspensions and solutions, and for water soluble powders, respectively), then an overall average was calculated (each type of oral formulation represented one “weight” in the global average).

The average daily dosages and doses were called Defined Daily Dosages for cattle in Canada (dddbovCA, in mg/kg of body weight per day) and Defined Daily Doses for cattle in Canada (DDDbovCA, in mg (or g)/animal per day), respectively. The average course dosages and doses were called Defined Course Dosages for cattle in Canada (dcdbovCA, in mg/kg of body weight per course) and Defined Course Doses for cattle in Canada (DCDbovCA, in mg (or g)/animal per course).

### Other Information Reported by AM and Route of Administration

The code in the Anatomical Therapeutic Chemical classification for veterinary medicinal products (ATCvet code) was searched in the ATCvet Index 2019 (41), and reported by product. The antimicrobial class and the category of the AM based on its importance in human medicine as defined by Health Canada (30) were also recorded for each AM. Four categories were described: category (I) AMs of “very high importance” (preferred option for treatment of serious human infections, without or with limited availability of alternative AMs); category (II) AMs of “high importance” (preferred option for treatment of serious human infections, but alternative AMs are available); category (III) AMs of “medium importance” (not the preferred option for treatment of serious human infections); and category (IV) AMs of “low importance” (AMs currently not used in human medicine).

## Results

Between April and December 2019, the status of 17 products (5 injectable products, 1 oral or intrauterine bolus, 7 water soluble powders, and 4 medicated premixes) changed from “marketed” to “dormant” in the DPD. The status of 8 products (4 injectable products, 2 medicated premixes, and 2 topical sprays) changed from “marketed” to “canceled post market.” These 25 products (Supplementary 1.1) were not used in the assignment of defined doses as they were not sold on December 2019 in Canada. No ophthalmic product was found with an indication for cattle. Eleven products (Supplementary 1.2) were excluded from calculations (9 subcutaneous implants containing oxytetracycline or tylosin, 1 medicated premix containing chlortetracycline and sulfamethazine and with only a growth promotion indication, and 1 medicated premix containing oxytetracycline and neomycin also with only a growth promotion indication).

A total of 131 products were retained for assignment of defined daily and course doses. For 101 and 30 products, the monograph was extracted from the DPD (Health Canada) and from the CVP, respectively. The last update of the monograph was <2 years for the DPD version, but generally was not indicated for the CVP version. The only combination of AMs that met the three criteria to be considered as one entity was the trimethoprim and sulfadoxine combination. This synergistic combination (42) was found in three injectable products at the fixed ratio of 1–5 (40 mg of trimethoprim and 200 mg of sulfadoxine per mL of injectable solution). Both trimethoprim and sulfadoxine were not found non-combined in any marketed products.

### Injectable Route

Thirty-nine injectable products were identified (detailed in Supplementary 2.1, 2.2). Average calculations by AM are detailed in Supplementary 2.3, and the summary is presented in Table 1. Antimicrobial agents found in injectable products were: ampicillin, benzylpenicillin (benzathine or procaine), ceftiofur, danofloxacin, enrofloxacin, florfenicol, gamithromycin, marbofloxacin, oxytetracycline, tildipirosin, tilmicosin, tulathromycin, tylosin, and the combination of sulfadoxine and trimethoprim. The combination of benzathine benzylpenicillin and procaine benzylpenicillin was found in one long-acting product at the fixed ratio of 1 for 1 (150,000 international units per mL for both salts). Procaine benzylpenicillin was also found alone in six other products. Benzathine and procaine benzylpenicillin are two prodrugs of benzylpenicillin not known to be synergistic; they were assigned separate defined doses. Products with both a single-dose therapy and a multiple-dose therapy contained danofloxacin (*n* = 1), enrofloxacin (*n* = 1), or florfenicol (*n* = 3). Products with a single-dose therapy only contained ceftiofur crystalline free acid (*n* = 1), gamithromycin (*n* = 1), marbofloxacin (*n* = 1), oxytetracycline dihydrate (*n* = 7), tildipirosin (*n* = 1), tilmicosin (*n* = 3), or tulathromycin (*n* = 1). For the products containing ceftiofur or oxytetracycline, the duration of effective concentration of the AM in plasma after administration was used to calculate a daily dose. For the products containing gamithromycin, marbofloxacin, tildipirosin, tilmicosin, or tulathromycin, the daily dose was estimated using the 7-day arbitrary duration of action. Antimicrobial agents identified in products authorized for lactating dairy cows as well as in products not for use in lactating dairy cows were: procaine benzylpenicillin, and oxytetracycline.

**Table 1:** Assignment of DDDbovCA and DCDbovCA values for antimicrobial agents used systemically through the injectable route for cattle in Canada.

**Antimicrobial agent**	**Combined with another antimicrobial agent in products?**	**Antimicrobial class**	**Category according to Health Canada**	**dddbovCA (mg per kg per day)**	**dcdbovCA (mg per kg per course)**	**DDDbovCA (g per animal per day)**	**DCDbovCA (g per animal per course)**
Ampicillin	No	Penicillins with extended spectrum	II	6.0	30.0	3.90	19.50
Benzylpenicillin (Penicillin G) Benzathine	Combined with benzylpenicillin procaine	Beta-lactamase sensitive penicillins	II	0.9	5.4	0.27	1.62
Benzylpenicillin (Penicillin G) Procaine	Combined with benzylpenicillin benzathine	Beta-lactamase sensitive penicillins	II	0.9	5.4	0.27	1.62
Benzylpenicillin (Penicillin G) Procaine	No	Beta-lactamase sensitive penicillins	II	8.8	40.2	4.96	20.88
Ceftiofur	No	Third-generation cephalosporins	I	1.2	6.0	0.80	3.92
Danofloxacin	No	Fluoroquinolones	I	3.0	10.0	0.90	3.00
Enrofloxacin	No	Fluoroquinolones	I	3.8	12.5	1.14	3.75
Florfenicol	No	Amphenicols	III	10.0	40.0	3.00	12.00
Gamithromycin	No	Macrolides	II	0.86	6.0	0.26	1.80
Marbofloxacin	No	Fluoroquinolones	I	1.4	10.0	0.43	3.00
Oxytetracycline	No	Tetracyclines	III	5.9	18.4	2.62	7.29
Tildipirosin	No	Macrolides	II	0.57	4.0	0.17	1.20
Tilmicosin	No	Macrolides	II	1.4	10.0	0.43	3.00
Trimethoprim and sulfadoxine combination	No	Combinations of sulfonamides and trimethoprim	II	16.0	64.0	10.40	41.60
Tulathromycin	No	Macrolides	II	0.36	2.5	0.11	0.75
Tylosin	No	Macrolides	II	17.6	70.4	5.28	21.12

*dddbovCA, Canadian Defined Daily Dosage for cattle (in mg/kg/day); DDDbovCA, Canadian Defined Daily Dose for cattle (in g/animal/day); dcdbovCA, Canadian Defined Course Dosage for cattle (in mg/kg/course); DCDbovCA, Canadian Defined Course Dose for cattle (in g/animal/course)*.

#### Oral Route

Fifty-two oral products other than medicated premixes were identified. Different types of formulations were available: boluses or tablets (12 products), suspensions or solutions (9 products), and water soluble powders (31 products). Twenty-seven, sixteen, and nine products contained one, two, and three AMs, respectively (detailed in Supplementary 3.1, 4.1, 4.2). Antimicrobial agents that could be found alone or in combination were neomycin, oxytetracycline, sulfamethazine, sulfapyridine, and tetracycline. Antimicrobial agents that were always found in combination in products were benzylpenicillin, streptomycin, succinylsulfathiazole, sulfaguanidine, sulfamerazine, sulfanilamide, and sulfathiazole. Sulfonamide-based products were numerous, and dosages varied widely from one product to another, depending on the type of sulfonamide, and the type of formulation. Aminoglycosides were reported under their sulfate form, and were kept as neomycin sulfate and streptomycin sulfate in calculations. Average calculations by AM are detailed in Supplementary 3.2 (AMs used non-combined) and Supplementary 4.3 (AMs used combined).

Twenty-three medicated premixes (detailed in Supplementary 3.3) were used for calculations and contained either ionophores (lasalocid, monensin, salinomycin), tetracyclines (chlortetracycline, oxytetracycline), or macrolides (tilmicosin, tylosin). No combination was identified. Only two products containing tilmicosin were designed for metaphylaxis purposes (reduction of morbidity in groups of feedlot beef cattle experiencing an outbreak of bovine respiratory disease). Other premixes were indicated for the prevention of diseases: foot rot (chlortetracycline), bacterial enteritis (chlortetracycline, oxytetracycline), liver abscesses (tylosin), and coccidiosis (lasalocid, monensin), or for growth promotion and feed efficiency (lasalocid, monensin, salinomycin).

The summary of defined daily and course dosages and doses for AMs used systemically through the oral route is presented in Table 2.

**Table 2:** Assignment of DDDbovCA and DCDbovCA values for antimicrobial agents used systemically through the oral route for cattle in Canada.

**Antimicrobial agent**	**Combined with another antimicrobial agent in products?**	**Antimicrobial class**	**Category according to Health Canada**	**dddbovCA (mg per kg per day)**	**dcdbovCA (mg per kg per course)**	**DDDbovCA (g per animal per day)**	**DCDbovCA (g per animal per course)**
**Antimicrobial agents used in oral products other than medicated premixes**							
Benzylpenicillin (Penicillin G)	Combined with streptomycin	Beta-lactamase sensitive penicillins	II	5.3	13.2	0.53	1.32
Monensin	No	Ionophores	IV	0.52	49.8	0.34	32.40
Neomycin sulfate	No	Aminoglycosides	II	16.3	48.8	1.63	4.88
Neomycin sulfate	Combined with sulfonamides or tetracyclines	Aminoglycosides	II	14.1	51.1	1.41	5.11
Oxytetracycline	No	Tetracyclines	III	12.3	48.3	2.43	10.23
Oxytetracycline	Combined with neomycin	Tetracyclines	III	12.7	57.0	1.27	5.70
Streptomycin sulfate	Combined with benzylpenicillin	Aminoglycosides	II	27.3	68.1	2.73	6.81
Succinylsulfathiazole	Combined with neomycin	Sulfonamides	III	57.6	144.0	5.76	14.40
Sulfaguanidine	Combined with neomycin and sulfathiazole	Sulfonamides	III	29.8	104.2	2.98	10.42
Sulfamerazine	Combined with sulfonamides	Sulfonamides	III	4.5	27.7	1.35	8.31
Sulfamethazine (Sulfadimidine)	No	Sulfonamides	III	101.6	406.1	30.47	121.85
Sulfamethazine (Sulfadimidine)	Combined with neomycin or sulfonamides	Sulfonamides	III	59.0	195.8	13.57	44.35
Sulfanilamide	Combined with sulfonamides	Sulfonamides	III	91.4	91.4	27.42	27.42
Sulfapyridine	No	Sulfonamides	III	179.2	537.5	53.76	161.25
Sulfapyridine	Combined with sulfonamides	Sulfonamides	III	24.8	99.1	7.44	29.73
Sulfathiazole	Combined with neomycin and sulfaguanidine, or sulfonamides	Sulfonamides	III	44.2	142.6	12.67	40.69
Tetracycline	No	Sulfonamides	III	10.4	60.0	1.04	6.00
Tetracycline	Combined with neomycin	Tetracyclines	III	13.3	60.0	1.33	6.00
**Antimicrobial agents used in medicated premixes**							
Chlortetracycline	No	Tetracyclines	III	0.66	59.4	0.09	8.10
Lasalocid	No	Ionophores	IV	0.89	79.8	0.27	23.94
Monensin	No	Ionophores	IV	0.53	47.5	0.21	18.77
Oxytetracycline	No	Tetracyclines	III	1.1	99.0	0.09	8.33
Salinomycin	No	Ionophores	IV	0.33	30.0	0.10	9.00
Tilmicosin	No	Macrolides	II	12.5	175.0	3.75	52.50
Tylosin	No	Macrolides	II	0.22	19.8	0.07	5.94

*dddbovCA, Canadian Defined Daily Dosage for cattle (in mg/kg/day); DDDbovCA, Canadian Defined Daily Dose for cattle (in g/animal/day); dcdbovCA, Canadian Defined Course Dosage for cattle (in mg/kg/course); DCDbovCA, Canadian Defined Course Dose for cattle (in g/animal/course). Dosages and doses were not determined for combined chlortetracycline and sulfamethazine (1 premix), and for combined neomycin sulfate and oxytetracycline (1 premix) (see Supplementary 1.2)*.

#### Intramammary, Intrauterine, and Topical Routes

Eight intramammary products (four for lactating cows, and four for dry cows) were authorized for use in Canada, all sold as 10-mL disposable single-use syringes (detailed in Supplementary 5.1). Three of the products indicated for lactating cows contained a single AM each (cefapirin, ceftiofur, or pirlimycin), and one product contained four AMs (procaine benzylpenicillin, dihydrostreptomycin, novobiocin, and polymyxin B). Three of the products indicated for dry cow therapy contained one AM each (cefapirin, ceftiofur, or cloxacillin), and one contained two AMs (procaine benzylpenicillin and novobiocin).

Five intrauterine products were authorized for use in Canada, marketed under different pharmaceutical formulations: disposable single-use syringes, injectable solutions, stable suspensions, and boluses (detailed in Supplementary 5.2). Three of them contained one AM each (cefapirin, gentamicin, or oxytetracycline). Two products contained a combination of two sulfonamides (sulfanilamide and sulfathiazole).

Four topical products were marketed in Canada for cattle (Detailed in Supplementary 5.3). Antimicrobial agents found in these products were: chlortetracycline, or a combination of two sulfonamides (sulfanilamide and sulfathiazole). Different formulations were available: sprays, creams, or powders.

The summary of defined daily and course doses for AMs used non-systemically is presented in Table 3.

**Table 3:** Assignment of DDDbovCA and DCDbovCA values for antimicrobial agents used non-systemically through the intramammary, intrauterine, and topical routes for cattle in Canada.

**Antimicrobial agent**	**Combined with another antimicrobial agent in products?**	**Antimicrobial class**	**Category according to Health Canada**	**DDDbovCA (mg per animal per day)**	**DCDbovCA (mg per animal per course)**
**Antimicrobial agents used through the intramammary route in cows during the lactation**					
Benzylpenicillin (Penicillin G) Procaine	Combined with dihydrostreptomycin, novobiocin, and polymyxin B sulfate	Beta-lactamase sensitive penicillins	II	60	90
Cefapirin	No	First-generation cephalosporins	II	400	400
Ceftiofur	No	Third-generation cephalosporins	I	125	250
Dihydrostreptomycin	Combined with benzylpenicillin procaine, novobiocin, and polymyxin B sulfate	Aminoglycosides	II	100	150
Novobiocin	Combined with benzylpenicillin procaine, dihydrostreptomycin, and polymyxin B sulfate	Aminocoumarins	Not categorized	150	225
Pirlimycin	No	Lincosamides	II	50	250
Polymyxin B sulfate	Combined with benzylpenicillin procaine, dihydrostreptomycin, and novobiocin	Polymyxins	I	6	9
**Antimicrobial agents used through the intramammary route in cows at drying-off**					
Benzylpenicillin (Penicillin G) procaine	Combined with novobiocin	Beta-lactamase sensitive penicillins	II	48	480
Cefapirin	No	First-generation cephalosporins	II	120	1,200
Ceftiofur	No	Third-generation cephalosporins	I	200	2,000
Cloxacillin	No	Beta-lactamase resistant penicillins	II	200	2,000
Novobiocin	Combined with benzylpenicillin procaine	Aminocoumarins	Not categorized	160	1,600
**Antimicrobial agents used through the intrauterine route in cows**					
Cefapirin	No	First-generation cephalosporins	II	500	500
Gentamicin sulfate	No	Aminoglycosides	II	200	200
Oxytetracycline	No	Tetracyclines	III	2,500	2,500
Sulfanilamide	Combined with sulfathiazole	Sulfonamides	III	2,880	2,880
Sulfathiazole	Combined with sulfanilamide	Sulfonamides	III	480	480
**Antimicrobial agents used through the topical route in cattle**					
Chlortetracycline	No	Tetracyclines	III	147	441
Sulfanilamide	Combined with sulfathiazole	Sulfonamides	III	444	2,220
Sulfathiazole	Combined with sulfanilamide	Sulfonamides	III	444	2,220

*DDDbovCA, Canadian Defined Daily Dose for cattle (in mg/animal/day); DCDbovCA, Canadian Defined Course Dose for cattle (in mg/animal/course)*.

## Discussion

Assignment of defined daily and course doses by species is an essential part of the evaluation of AMU. The main benefit of using dose-based metrics for AMU quantification is the ability to compare between different AMs, species, and regions, as it is the only metric that accounts for dose differences (and then for differences in animal weights). We used a reproducible method to assign DDDbovCA and DCDbovCA values for all AM currently used for cattle in Canada. This method will allow easy updates in the future to include new products in the calculations, or remove products that are no more sold.

DDDs were first described by the WHO in the seventies (43), and were aimed at providing an international measure system to quantify active substances found in human medicines. The WHO Collaborating Center for Drug Statistics Methodology updates annually their guidelines for DDD assignment (23). The DDDs are not intended to correspond perfectly to each regional specific usage of AMs, but with an internationally accepted metric, comparisons of AMU between regions in the world are easily performed.

A larger amount of long-acting veterinary medicines are available in comparison with human medicines. This observation explains the emergence of another unit for veterinary products: the defined course dose, first developed by the French Agency for Food, Environmental and Occupational Health & Safety (44) as ACD (Animal Course Dose), and adapted by the EMA in the ESVAC project as DCDvet (Defined Course Dose for Animals). The DDDvet (Defined Daily Dose for animals) and DCDvet values were assigned in 2016 (22) based on doses from nine European countries for cattle, poultry, and swine, and are now used for comparison of AMU in Europe (8). Applying these values to Canadian AMU data, however, is very difficult because of notable differences between Europe and Canada, both in the types of AMs used, and in the doses they are used at.

This study highlighted differences between Europe and Canada in terms of AMs marketed: 2 (out of 8) intramammary products and 3 (out of 5) intrauterine products available in Canada had no equivalent in Europe. Three (out of 7) AMs from medicated premixes and 7 (out of 13) oral AMs (other than premixes) were sold in Canada but not identified in Europe. The three AMs identified in Canadian medicated premixes but not in Europe were ionophores (lasalocid, monensin, and salinomycin) that are categorized as antimicrobials by Health Canada. All injectable AMs available in Canada were also listed in ESVAC reports. Moreover, for systemically-used AMs (injectable or oral), the comparison between European and Canadian daily doses showed that 74% of Canadian doses were lower than European doses (relative difference inferior by more than 10%), 11% of doses were relatively similar between Europe and Canada (relative difference between −10% and +10%), and 15% of Canadian doses were greater than European doses (relative difference superior by more than 10%). Because of the lower doses in general for Canada in comparison with Europe, and because the AMs were considered separately when identified in combinations, for an equal weight of AMs, the Canadian measure system will report a higher dose-based AMU (i.e., a higher number of DDDbovCA or DCDbovCA).

Main calculation differences between Europe and Canada concerned the oral products: we did separate medicated premixes from other oral formulations as it was assumed that their usage was really different (mass medication vs. individual treatment, duration of administration, type of cattle targeted by the medication). This was easily performed as most of the AMs found in premixes were different than AMs found in other oral formulations, with the exceptions of monensin and oxytetracycline that were identified in both premixes, and tablets (monensin), or soluble powders (oxytetracycline). Among oral formulations, AMs found in combinations were assigned separate DDDbovCA and DCDbovCA values than non-combined AMs. This latter decision was also different from that of ESVAC which used the same DDDvet and DCDvet values for an AM identified in oral combinations vs. oral single forms (36).

In injectable products, only two combinations were identified: combined trimethoprim and sulfadoxine, and combined procaine benzylpenicillin and benzathine benzylpenicillin. The combined trimethoprim and sulfadoxine in our system was the only combination that was kept undivided in assignment of defined doses. This combination is known to be synergistic because both AMs involve sequential inhibition of successive steps in the folate metabolism. Its usage as a combination is recommended instead of using just the trimethoprim or the sulfadoxine part (42).

Calculations were achieved by making some arbitrary decisions in order to propose defined doses for every AM marketed currently in Canada. The following decisions could be seen as limitations: need to use approximations of standard body weights, average daily intake (food, water), average cattle targeted by the label (beef/dairy, young/adult), and even approximation of the duration of action for long-acting products. Body weights and daily requirements vary depending on the age, sex, production type, and metabolic status of the animal. However, defining approximations was essential to obtain doses for every product. Three standard body weights were defined for systemically-used AMs: 650 kg for injectable products authorized for lactating cows, 300 kg for injectable products not for use in lactating cows and for oral products labeled for all types of cattle, and 100 kg for oral products labeled specifically for calves. The standard weights defined for Canada differ from the weights available from the ESVAC publications [425 kg for an adult cattle, 200 kg for a heifer, and 140 kg for a young cattle; Table A14 in European Medicines Agency, European Surveillance of Veterinary Antimicrobial Consumption (8)]. Nevertheless, in the current article, dosages (mg/kg) and doses (g/animal) were reported for systemically-used AMs. Reporting the total dose per animal is innovative as other publications generally only present dosages (22). Different standard weights could be applied to the dosages presented in this paper in order to obtain another set of doses more relevant for a specific context or to allow more direct comparisons with other countries.

Several durations of action were also defined: 10 days for dry-cow products, 24 h for intrauterine products, and 7 days for long-acting injectable macrolides and fluoroquinolones (when no other information was identified from the product monograph). These latter periods are not intended to be representative of the exact true duration of action for each product. They are approximations and were defined strictly for allowing assignment of daily doses for long-acting products in a transparent way. The ESVAC project did not assign doses for parenteral gamithromycin (daily and course), parenteral tildipirosin (daily and course), and dry-cow products (daily) (22). Without defined doses, these specific products are not quantified in reports using the DDDvet unit. One of our objectives was to propose defined doses for all AMs without exception in order to include them in reports on AMU using a daily-based indicator.

Defined doses are technical units; they are not intended to reflect recommended doses or to approximate actual doses. As an example, more than 80% of Canadian dairy producers reported off-label treatment for clinical mastitis (longer duration or higher frequency) in a recent study (45).

With an objective of harmonization between countries, the next step in assignment of veterinary defined doses would be to have just one set of defined values that could be used worldwide. Because the world market of antibiotics is not stable over time (new release of products, cessation of the sales of some products, etc.), defined doses should be updated regularly, as the WHO does for human drugs.

DDDbovCA and DCDbovCA can now be used to report on AMU in cattle in Canada. In the future, there will be great interest to compare defined vs. used and prescribed doses for the different Canadian provinces.

## Data Availability Statement

All datasets generated for this study are included in the article/Supplementary Material.

## Author Contributions

HL, SD, and DF contributed to the conception and design of the study. HL wrote the first draft of the manuscript. All authors listed have made a substantial, direct and intellectual contribution to the work, and approved the submitted version.

### Conflict of Interest

The authors declare that the research was conducted in the absence of any commercial or financial relationships that could be construed as a potential conflict of interest.
