# Supplementary material for: Assignment of Canadian Defined Daily Doses and Canadian Defined Course Doses for Quantification of Antimicrobial Usage in Cattle
Source: Front Vet Sci. 2020 Jan 29;7:10. doi: 10.3389/fvets.2020.00010 (PMC7001643; doi:10.3389/fvets.2020.00010)
Supplement: Supplementary file 1 [file Data_Sheet_1.docx]

**Supplementary Material – Table of contents**

**1. Details of the products excluded from calculations.**

Supplementary 1.1. Details of the 25 products for which the status in the Drug Product Database (Health Canada) changed from “marketed” to “dormant” or “cancelled post market” between April and December 2019.

Supplementary 1.2. Details of the 11 products marketed in Canada, and excluded from calculations.

**2. Details of the injectable products.**

Supplementary 2.1. Details of the 16 injectable products authorized for lactating dairy cows, containing at least one antimicrobial agent, and marketed in Canada with at least one indication for cattle.

Supplementary 2.2. Details of the 23 injectable products not for use in lactating dairy cows, containing at least one antimicrobial agent, and marketed in Canada with at least one indication for cattle.

Supplementary 2.3. Average daily dosage, course dosage, daily dose, and course dose, for procaine benzylpenicillin (not combined), ceftiofur, and oxytetracycline (from injectable products).

**3. Details of the oral products containing exactly one antimicrobial agent.**

Supplementary 3.1. Details of the 27 oral products other than medicated premixes, containing exactly one antimicrobial agent, and marketed in Canada with at least one indication for cattle.

Supplementary 3.2. Average daily dosage, course dosage, daily dose, and course dose, for oxytetracycline, sulfamethazine (sulfadimidine), and tetracycline (from oral products other than medicated premixes, containing exactly one antimicrobial agent).

Supplementary 3.3. Details of the 23 medicated premixes containing exactly one antimicrobial agent, and marketed in Canada with at least one indication for cattle.

**4. Details of the oral products containing more than one antimicrobial agent.**

Supplementary 4.1. Details of the 16 oral products other than medicated premixes, containing exactly two antimicrobial agents, and marketed in Canada with at least one indication for cattle.

Supplementary 4.2. Details of the 9 oral products other than medicated premixes, containing exactly three antimicrobial agents, and marketed in Canada with at least one indication for cattle.

Supplementary 4.3. Average daily dosage, course dosage, daily dose, and course dose, for neomycin sulfate, oxytetracycline, sulfamerazine, sulfamethazine (sulfadimidine), and sulfathiazole (from oral products other than medicated premixes, containing more than one antimicrobial agent).

**5. Details of the intramammary, intrauterine, and topical products.**

Supplementary 5.1. Details of the 8 intramammary products containing at least one antimicrobial agent, and marketed for cattle in Canada.

Supplementary 5.2. Details of the 5 intrauterine products containing at least one antimicrobial agent, and marketed in Canada with at least one indication for cattle.

Supplementary 5.3. Details of the 4 topical products containing at least one antimicrobial agent, and marketed in Canada with at least one indication for cattle.

**Supplementary 1.1. Details of the 25 products for which the status* in the Drug Product Database (Health Canada) changed from “marketed” to “dormant” or “cancelled post market” between April and December 2019.**

Legend: **DIN**: Drug Identification Number (computer-generated eight-digit number assigned by Health Canada to a drug product prior to being marketed in Canada).

| **DIN of the product** | **Antimicrobial agent(s) contained in the product** | **Route of administration** | **Status* in December 2019 in the Drug Product database (Health Canada)** |
| --- | --- | --- | --- |
| 02030551 | Penicillin G Benzathine and Penicillin G Procaine | Injectable | Dormant |
| 02059991 | Penicillin G Procaine | Injectable | Dormant |
| 02239151 | Penicillin G Procaine | Injectable | Cancelled Post Market |
| 00544051 | Penicillin G Procaine | Injectable | Cancelled Post Market |
| 02208210 | Oxytetracycline Hydrochloride | Injectable | Dormant |
| 02259958 | Oxytetracycline Dihydrate | Injectable | Dormant |
| 02248883 | Oxytetracycline Hydrochloride | Injectable | Cancelled Post Market |
| 02287080 | Oxytetracycline Dihydrate | Injectable | Cancelled Post Market |
| 02145995 | Trimethoprim and Sulfadoxine | Injectable | Dormant |
| 02010054 | Tetracycline Hydrochloride | Oral and Intrauterine (bolus) | Dormant |
| 01943251 | Neomycin Sulfate and Oxytetracycline Hydrochloride | Oral (powder for solution) | Dormant |
| 00780782 | Oxytetracycline Hydrochloride | Oral (powder for solution) | Dormant |
| 01991183 | Tetracycline Hydrochloride | Oral (powder for solution) | Dormant |
| 00786098 | Tetracycline Hydrochloride | Oral (powder for solution) | Dormant |
| 00861995 | Tetracycline Hydrochloride | Oral (powder for solution) | Dormant |
| 00896535 | Tetracycline Hydrochloride | Oral (powder for solution) | Dormant |
| 01971530 | Sulfamethazine and Sulfathiazole | Oral (powder for solution) | Dormant |
| 00685224 | Oxytetracycline Hydrochloride | Drug Premix | Dormant |
| 00780774 | Oxytetracycline Hydrochloride | Drug Premix | Dormant |
| 02223902 | Oxytetracycline Hydrochloride | Drug Premix | Dormant |
| 02242693 | Chlortetracycline Hydrochloride | Drug Premix | Dormant |
| 02242816 | Chlortetracycline Hydrochloride and Sulfamethazine | Drug Premix | Cancelled Post Market |
| 02337215 | Chlortetracycline Hydrochloride and Sulfamethazine | Drug Premix | Cancelled Post Market |
| 00486388 | Neomycin Sulfate (and Gentian Violet^1^) | Topical (spray) | Cancelled Post Market |
| 02407345 | Neomycin Sulfate (and Gentian Violet^1^) | Topical (spray) | Cancelled Post Market |

*A “marketed” status refers to an active DIN that is currently being sold in Canada. A “dormant” status refers to an active DIN that was previously marketed in Canada but for which there have been no sales for a period of at least 12 months. A “cancelled post market” status refers to a DIN that is cancelled further to the discontinuation of the sale by the manufacturer pursuant to Section C.01.014.6 (1) (a) of the Food and Drug Regulations.

^1^Due to the potential risk of cancer associated with gentian violet, Health Canada worked with all manufacturers with licensed drug products containing gentian violet to remove them from the market (June 2019; <https://healthycanadians.gc.ca/recall-alert-rappel-avis/hc-sc/2019/70179a-eng.php>).

**Supplementary 1.2. Details of the 11 products marketed in Canada, and excluded from calculations.**

Legend: **DIN**: Drug Identification Number (computer-generated eight-digit number assigned by Health Canada to a drug product prior to being marketed in Canada).

| **DIN of the product** | **Type of formulation** | **Antimicrobial agent(s) contained in the product** | **Strength of active antimicrobial agent(s) in the product** | **Rational for exclusion from calculations** |
| --- | --- | --- | --- | --- |
| 00590932 | Subcutaneous hormonal implant | Oxytetracycline | 25.7 mg estradiol per implant; coated with not less than 0.5 mg oxytetracycline | Antimicrobial agent included as a local antibacterial for reducing the incidence of abscess formation at the implant site |
| 02245743 | Subcutaneous hormonal implant | Tylosin Tartrate | Each dose of 9 pellets consists of 8 pellets containing a total of 200 mg of progesterone USP and 20 mg estradiol benzoate plus 1 pellet containing 29 mg tylosin tartrate. |  |
| 02245744 | Subcutaneous hormonal implant | Tylosin Tartrate | Each dose of 5 pellets consists of 4 pellets containing a total of 100 mg of progesterone USP and 10 mg estradiol benzoate plus 1 pellet containing 29 mg tylosin tartrate. |  |
| 02245746 | Subcutaneous hormonal implant | Tylosin Tartrate | Each dose of 9 pellets consists of 8 pellets containing a total of 200 mg of testosterone propionate USP and 20 mg estradiol benzoate plus 1 pellet containing 29 mg tylosin tartrate. |  |
| 02407760 | Subcutaneous hormonal implant | Tylosin Tartrate | Implant consisting of 10 small yellow pellets containing 200 mg of trenbolone acetate USP and 20 mg of estradiol USP, and one blue pellet containing 29 mg of tylosin tartrate |  |
| 02449803 | Subcutaneous hormonal implant | Tylosin Tartrate | Implant consisting of 2 small yellow pellets containing 40 mg of trenbolone acetate USP and 8 mg of estradiol USP, and one blue pellet containing 29 mg of tylosin tartrate |  |
| 02245665 | Subcutaneous hormonal implant | Tylosin Tartrate | Implant containing 140 mg of trenbolone acetate, 14 mg of estradiol USP and 29 mg of tylosin tartrate. |  |
| 02245745 | Subcutaneous hormonal implant | Tylosin Tartrate | Implant containing 120 mg of trenbolone acetate, 24 mg of estradiol USP and 29 mg of tylosin tartrate. |  |
| 02366916 | Subcutaneous hormonal implant | Tylosin Tartrate | Implant containing 100 mg of trenbolone acetate, 10 mg of estradiol USP and 29 mg of tylosin tartrate |  |
| 00655708 | Medicated premix | Chlortetracycline and Sulfamethazine | Drug premix containing 77 g/kg chlortetracycline hydrochloride and 77 g/kg sulfamethazine. | Medically Important Antimicrobials^1^ used as an aid in the maintenance of weight gains and feed efficiency in beef cattle during periods of stress due to weaning, shipping or handling. |
| 02325098 | Medicated premix | Oxytetracycline and Neomycin Sulfate | Drug premix containing 110 g/kg oxytetracycline hydrochloride and 110 g/kg neomycin sulfate. |  |

^1^Health Canada is removing growth promotion claims and related directions for use from Medically Important Antimicrobials (MIAs) drug labels (<https://www.canada.ca/en/public-health/services/antibiotic-antimicrobial-resistance/animals/actions/responsible-use-antimicrobials.htm>). The MIAs are antimicrobials belonging to the Categories I, II, and III according to Health Canada’ Categorization of Antimicrobial Drugs Based on Importance in Human Medicine (<https://www.canada.ca/en/health-canada/services/drugs-health-products/veterinary-drugs/antimicrobial-resistance/categorization-antimicrobial-drugs-based-importance-human-medicine.html>).

**Supplementary 2.1. Details of the 16 injectable products authorized for lactating dairy cows, containing at least one antimicrobial agent, and marketed in Canada with at least one indication for cattle.**

Legend: **ATCvet**: Anatomical Therapeutic Chemical Classification System for veterinary medicinal products; **BRD**: Bovine Respiratory Disease(s); **DIN**: Drug Identification Number; **IU**: International Unit(s); **MIC**: Minimum Inhibitory Concentration.

| **DIN of products marketed in Canada** | **Antimicrobial agent(s)** | **Indication(s)** | **Product Monograph** | **Interpretations from the product monograph** | **Daily dosage (mg/kg/day)** | **Course dosage (mg/kg/course)** | **Daily dose (mg/animal/day)** | **Course dose (mg/animal/course)** | **ATCvet Code** |
| --- | --- | --- | --- | --- | --- | --- | --- | --- | --- |
| 00849405 | Ampicillin | Bacterial pneumonia, shipping fever complex and enteritis caused by *Staphylococcus* sp and *Escherichia coli* susceptible to ampicillin. Also indicated for surgical prophylaxis. | The dosage is 6 mg/kg of body weight once daily by intramuscular injection. Treatment should be continued for 48 to 72 hours after the animal has become afebrile or asymptomatic. Do not treat cattle for more than 7 days. | The average duration of treatment is 5 days (3 to 7 days). The daily dosage is **6** mg/kg/day. The course dosage is 6*5 = **30** mg/kg/course. | **6.0** | **30** | 6*650 = **3,900** | 30*650 = **19,500** | **QJ01CA01** (Antibacterials for systemic use – beta-lactam antibacterials, penicillins with extended spectrum) |
| 01983253  02245714  02267071  02408864 | Benzylpenicillin Procaine | As an aid in the treatment of the following infections caused by bacteria susceptible to penicillin: bacterial pneumonia, calf diphtheria, foot rot, metritis, wound infections. | 21,000 IU/kg of body weight intramuscularly once daily until 2 days after clinical signs disappear. Do not exceed 5 days of treatment. | The average duration of treatment is 4 days (3 to 5 days). The course dosage is 21000*0.0006*4 = **50.4** mg/kg/course. | **12.6** | **50.4** | 12.6*650 = **8,190** | 50.4*650 = **32,760** | **QJ01CE09** (Antibacterials for systemic use – beta-lactam antibacterials, beta-lactamase sensitive penicillins) |
| 00491020 | Benzylpenicillin Procaine | For the treatment of shipping fever syndrome, wound infections, navel infections, foot rot, bacterial infections associated with pneumonia, as well as for bronchitis and tracheitis when caused by or associated with penicillin susceptible organisms. | Inject deep into the muscle every 24 hours. Continue treatment for 1-2 days after symptoms disappear. If no improvement is noted within 36-48 hours re-evaluate the diagnosis. Give 6,000 IU per kg of body weight. Increase the dose to 12,000 IU – 18,000 IU per kg for serious infection. | The duration of treatment is unknown. The daily dosage is (6000+18000)/2 = 12000 UI/kg/day, or 12000*0.0006 = **7.2** mg/kg/day. | **7.2** | Not determined | 7.2*650 = **4,680** | Not determined | **QJ01CE09** (Antibacterials for systemic use – beta-lactam antibacterials, beta-lactamase sensitive penicillins) |
| 00813567  02458861 | Ceftiofur (as Sodium for 00813567 / as Hydrochloride for 02458861) | For the **treatment of BRD** (shipping fever, pneumonia) associated with *Mannheimia haemolytica*, *Pasteurella multocida* and *Haemophilus somnus*. For the **treatment of acute bovine interdigital necrobacillosis** (foot rot, pododermatitis) associated with *Fusobacterium necrophorum* and *Bacteroides melaninogenicus*. | Administer by intramuscular (00813567) / subcutaneous (02458861) injection at the dosage of 1.0 mg ceftiofur per kg of body weight. Treatment should be repeated every 24 hours for a total of 3 treatments. Additional treatments may be administered on days 4 and 5 for animals which do not show a satisfactory response (not recovered) after the initial 3 treatments. | The average duration of treatment is 4 days (3 to 5 days). The daily dosage is **1** mg/kg/day and the course dosage is **4** mg/kg/course. | **1.0** | **4.0** | 1*650 = **650** | 4*650 = **2,600** | **QJ01DD90** (Antibacterials for systemic use – other beta-lactam antibacterials, third-generation cephalosporins) |
| 02317214  02417677  02443260 | Ceftiofur (as Hydrochloride) | For the **treatment of BRD** (shipping fever, pneumonia) associated with *Mannheimia haemolytica*, *Pasteurella multocida* and *Histophilus somni*. For the **treatment of acute bovine interdigital necrobacillosis** (foot rot, pododermatitis) associated with *Fusobacterium necrophorum* and *Bacteroides melaninogenicus*. For the **treatment of acute post-partum metritis** commonly associated with *Arcanobacterium pyogenes*, *Escherichia coli* and *Fusobacterium necrophorum*. | • **For BRD and acute bovine interdigital necrobacillosis**: Administer by intramuscular or subcutaneous injection 1.0 mg ceftiofur equivalents per kg of body weight. Treatment should be repeated every 24 hours for a total of 3 treatments. Additional treatments may be administered on days 4 and 5 for animals which do not show a satisfactory response (not recovered) after the initial 3 treatments.  • **For acute post-partum metritis**: Administer by intramuscular or subcutaneous injection at a dosage of 2.2 mg ceftiofur per kg of body weight. Treatment should be repeated every 24 hours for a total of five treatments. | • **For BRD and acute bovine interdigital necrobacillosis**: The average duration of treatment is 4 days (3 to 5 days). The daily dosage is **1** mg/kg/day and the course dosage is **4** mg/kg/course.  • **For acute post-partum metritis**: The duration of treatment is 5 days. The daily dosage is **2.2** mg/kg/day and the course dosage is 2.2*5 = **11** mg/kg/course. | (1+2.2)/2 = **1.6** | (4+11)/2 = **7.5** | 1.6*650 = **1,040** | 7.5*650 = **4,875** | **QJ01DD90** (Antibacterials for systemic use – other beta-lactam antibacterials, third-generation cephalosporins) |
| 02360586 | Ceftiofur (as Crystalline Free Acid) | For the **treatment of BRD** associated with *Mannheimia haemolytica*, *Pasteurella multocida* and *Haemophilus somnus*. For the **treatment of bovine foot rot** (interdigital necrobacillosis) associated with *Fusobacterium necrophorum* and *Porphyromonas levii*. | Administer a **single dose of 6.6 mg ceftiofur equivalents per kg body weigh** by subcutaneous injection in the base of the ear only. Administration of ceftiofur to cattle as ceftiofur crystalline free acid provides effective concentrations of ceftiofur and desfuroylceftiofur-related metabolites in plasma above the **MIC90** for BRD label pathogens *Pasteurella multocida*, *Mannheimia haemolytica* and *Histophilus somni* for generally not less than 150 hours (**6.25 days**) after a single administration. | The duration of action is expected to be **6.25 days** based on effective concentrations in plasma.  The course dosage is **6.6** mg/kg/course and the daily dosage is estimated to be 6.6/6.25 = **1.056** mg/kg/day. | **1.1** | **6.6** | 1.1*650 = **715** | 6.6*650 = **4,290** | **QJ01DD90** (Antibacterials for systemic use – other beta-lactam antibacterials, third-generation cephalosporins) |
| 02184664 | Oxytetracycline (as Hydrochloride) | For the treatment of bacterial pneumonia, pasteurellosis (associated with shipping fever complex), mastitis, metritis, calf scours (bacterial enteritis), foot rot, navel ill, calf diphtheria, leptospirosis, blackleg/malignant edema, peritonitis and joint ill. | Administer intramuscularly or intravenously at the rate of 3 mL (100 mg of oxytetracycline hydrochloride per mL) per 45 kg of body weight per day for 2 to 3 days. | The average duration of treatment is 2.5 days. The daily dosage is 3*100/45 ≈ **6.7** mg/kg/day. The course dosage is 3*100*2.5/45 ≈ **16.7** mg/kg/course. | **6.7** | **16.7** | 6.7*650 = **4,355** | 16.7*650 = **10,855** | **QJ01AA06** (Antibacterials for systemic use – tetracyclines) |
| 00555657  01923838  02184559 | Trimethoprim and Sulfadoxine combination | For the treatment of respiratory tract infections (bacterial pneumonias including bovine pneumonic pasteurellosis – shipping fever), alimentary tract infections (primarily enteric and septicaemic colibacillosis and salmonellosis), infectious pododermatitis (foot rot, foul in the foot) and septicaemias. | Should be administered at a dose rate of 16 mg/kg body weight daily. Intramuscular injection is recommended for cattle, but if a particularly rapid response is required in acute infections, the product can be administered by slow intravenous injection. Treatment should continue for 2-3 days after symptoms have subsided. The usual course of treatment is for not longer than 5 consecutive days. | The average duration of treatment is 4 days (3 to 5 days). The daily dosage is **16** mg/kg/day. The course dosage is 16*4 = **64** mg/kg/course. | **16** | **64** | 16*650 = **10,400** | 64*650 = **41,600** | **QJ01EW13** (Antibacterials for systemic use – combinations of sulfonamides and trimethoprim, incl. derivatives) |

**Supplementary 2.2. Details of the 23 injectable products not for use in lactating dairy cows, containing at least one antimicrobial agent, and marketed in Canada with at least one indication for cattle.**

Legend: **ATCvet**: Anatomical Therapeutic Chemical Classification System for veterinary medicinal products; **BRD**: Bovine Respiratory Disease(s); **DIN**: Drug Identification Number; **IBK**: Infectious Bovine Keratoconjunctivitis; **IU**: International Unit(s); **MIC**: Minimum Inhibitory Concentration.

| **DIN of products marketed in Canada** | **Antimicrobial agent(s)** | **Indication(s)** | **Product Monograph** | **Type of cattle targeted by the product** | **Interpretations from the product monograph** | **Daily dosage (mg/kg/day)** | **Course dosage (mg/kg/course)** | **Daily dose (mg/animal/day)** | **Course dose (mg/animal/course)** | **ATCvet Code** |
| --- | --- | --- | --- | --- | --- | --- | --- | --- | --- | --- |
| 01983377 | Benzylpenicillin Benzathine | As an aid in the treatment of local and systemic infections caused by bacteria including blackleg, foot rot, metritis and shipping fever complex in cattle. | Should be given by deep intramuscular injection, 6000 IU each of benzylpenicillin procaine and benzylpenicillin benzathine per kg of body weight. Repeat the dose in 3 to 5 days if required.  Interpretation: The course dosage is either 6000 IU (3.6 mg) or 12000 IU (7.2 mg) each of benzylpenicillin procaine and benzylpenicillin benzathine. The average duration of action after one injection is 4 days. | Do not use in lactating dairy cows. | The average course dosage is (3.6+7.2)/2 = **5.4** mg/kg/course. The average daily dosage is 3.6/4 = **0.9** mg/kg/day. | **0.90** | **5.4** | 0.9*300 = **270** | 5.4*300 = **1,620** | **QJ01CR50** (Antibacterials for systemic use – beta-lactam antibacterials, combinations of penicillins) |
|  | Benzylpenicillin Procaine |  |  |  | The average course dosage is (3.6+7.2)/2 = **5.4** mg/kg/course. The average daily dosage is 3.6/4 = **0.9** mg/kg/day. | **0.90** | **5.4** | 0.9*300 = **270** | 5.4*300 = **1,620** |  |
| 02227320 | Benzylpenicillin Procaine | For the treatment of bacterial pneumonia, calf diphtheria, foot rot, metritis, and wound infections. | For intramuscular and subcutaneous administration in non-lactating cattle. The recommended dose rate is 20 mg procaine penicillin per kg bodyweight. If necessary, a second dose may be administered after 72 hours. | Do not administer to lactating dairy cattle. | The course dosage is either 20 or 40 mg/kg/course, for an average of **30** mg/kg/course. The daily dosage is 20/3 ≈ **6.7** mg/kg/day. | **6.7** | **30** | 6.7*300 = **2,010** | 30*300 = **9,000** | **QJ01CE09** (Antibacterials for systemic use – beta-lactam antibacterials, beta-lactamase sensitive penicillins) |
| 02261200 | Danofloxacin | For the **treatment of BRD** associated with *Mannheimia haemolytica* and *Pasteurella multocida*. | Single-Dose Therapy: Administer subcutaneously at 8 mg/kg of body weight as a one-time injection.  Multi-Day Therapy: Administer subcutaneously at 6 mg/kg of body weight with this treatment repeated once approximately 48 hours following the first injection | Do not use in dairy cattle. Do not use in veal calves. | Single-Dose Therapy: The course dosage is **8** mg/kg/day.  Multi-Day Therapy: The course dosage is **12** mg/kg. The daily dosage is 6/2 = **3** mg/kg. | **3.0** | (8+12)/2 = **10** | 3*300 = **900** | 10*300 = **3,000** | **QJ01MA92** (Antibacterials for systemic use – fluoroquinolones) |
| 02249243 | Enrofloxacin | For the **treatment of BRD** associated with *Mannheimia haemolytica* and  *Pasteurella multocida*. | Single-Dose Therapy: Administer once, a subcutaneous dose of 7.5 - 12.5 mg/kg of body weight.  Multiple-Dose Therapy: Administer daily for 3 days, a subcutaneous dose of 2.5 - 5.0 mg/kg of body weight. For animals that are clinically improved but still exhibit some signs of disease, additional treatments may be given on days four and five. | Do not use in female dairy cattle 20 months of age or older. Do not use in veal calves. | Single-Dose Therapy: The course dosage is (7.5+12.5)/2 = **10** mg/kg/course.  Multiple-Dose Therapy: The average duration of treatment is 4 days (3 to 5 days). The daily dosage is (2.5+5.0)/2 = **3.75** mg/kg and the course dosage is 3.75*4 = **15** mg/kg. | **3.8** | (10+15)/2 = **12.5** | 3.8*300 = **1,140** | 12.5*300 = **3,750** | **QJ01MA90** (Antibacterials for systemic use – fluoroquinolones) |
| 02216558  02458527 | Florfenicol | For the **treatment of BRD complex**, also called shipping fever, associated with *Mannheimia haemolytica*, *Pasteurella multocida* and *Histophilus somni*. For the **reduction of morbidity associated with BRD** in feedlot calves, caused by *Mannheimia haemolytica*, *Pasteurella multocida* and *Histophilus somni*, during the first 28 days in the feedlot, when administered at the time of arrival. For the **treatment of bovine interdigital phlegmon** (foot rot, interdigital necrobacillosis, infectious pododermatitis) associated with *Fusobacterium necrophorum* and *Bacteroides melaninogenicus*. For the **treatment of IBK** (Pinkeye) caused by *Moraxella bovis*. | Should be administered to cattle **either by a single subcutaneous injection** at a dose rate of 40 mg/kg body weight; **or by intramuscular injection** at a dose of 20 mg/kg body weight, a total of two doses with a 48 hour interval should be given. For feedlot calves at high risk of developing bovine respiratory disease, only the subcutaneous route of administration (40 mg/kg, once on arrival in the feedlot) is recommended. | Not for use in dairy cows 20 months of age or older. Not for use in veal calves. | Single-Dose Therapy: The course dosage is **40** mg/kg/course.  Multiple-Dose Therapy: The course dosage is 2*20 = **40** mg/kg/course. The daily dosage is 20/2 = **10** mg/kg/day. | **10** | **40** | 10*300 = **3,000** | 40*300 = **12,000** | **QJ01BA90** (Antibacterials for systemic use – amphenicols) |
| 02330997 | Florfenicol | For the **treatment of BRD complex** associated with *Mannheimia haemolytica*, *Pasteurella multocida* and *Histophilus somni* and BRD associated pyrexia. | Should be administered **as a single subcutaneous dose** of 40 mg/kg florfenicol and 2.2 mg/kg flunixin. | Not for use in cattle of breeding age. Not for use in lactating and dry dairy cows. | The duration of action is unknown. The course dosage is **40** mg/kg/course. | Not determined | **40** | Not determined | 40*300 = **12,000** | **QJ01BA90** (Antibacterials for systemic use – amphenicols) |
| 02347407 | Gamithromycin | For the **treatment of BRD** associated with *Mannheimia haemolytica*, *Pasteurella multocida*, *Histophilus somni* and *Mycoplasma bovis*. For the **reduction of morbidity associated with BRD** in feedlot calves, caused by *Mannheimia haemolytica*, *Pasteurella multocida* and *Histophilus somni*, during the first 10 days in the feedlot, when administered at the time of arrival. | Administer **a single subcutaneous injection** in the neck at a dose of 6 mg gamithromycin/kg body weight. Most animals will respond to treatment within 3 to 5 days. | Do not use in lactating dairy cattle and in gestating cows or heifers, which are intended to produce milk for human consumption, within 2 months of expected parturition. | The course dosage is **6** mg/kg/course. The daily dosage is defined as 6/7 ≈ **0.86** mg/kg/day. | **0.86** | **6.0** | 6*300/7 ≈ **257** | 6*300 = **1,800** | **QJ01FA95** (Antibacterials for systemic use – macrolides, lincosamides and streptogramins) |
| 02474441 | Marbofloxacin | For the individual **treatment of BRD** associated with susceptible strains of *Mannheimia hæmolytica* and *Pasteurella multocida* in affected cattle. | The recommended dosage is 10 mg/kg body weight in a **single intramuscular injection**. | Do not use in dairy cattle and in calves to be processed for veal. | The course dosage is **10** mg/kg/course. The daily dosage is defined as 10/7 ≈ **1.4** mg/kg/day. | **1.4** | **10** | 10*300/7 ≈ **429** | 10*300 = **3,000** | **QJ01MA93** (Antibacterials for systemic use – fluoroquinolones) |
| 00616311 | Oxytetracycline (as Hydrochloride) | For use in the treatment of bacterial enteritis (scours), bacterial pneumonia, blackleg/malignant edema, calf diphtheria, foot rot, joint ill, leptospirosis, mastitis, metritis, navel ill, pasteurellosis (associated with shipping fever complex). | Administer by intravenous or intramuscular injection at the rate of 3 mL (100 mg of oxytetracycline hydrochloride per mL) per 45 kg body weight once daily for 2 to 3 days. | This product must not be used in lactating dairy cattle. | The average duration of treatment is 2.5 days. The daily dosage is 3*100/45 ≈ **6.7** mg/kg/day. The course dosage is 3*100*2.5/45 ≈ **16.7** mg/kg/course. | **6.7** | **16.7** | 6.7*300 = **2,010** | 16.7*300 = **5,010** | **QJ01AA06** (Antibacterials for systemic use – tetracyclines) |
| 00670804  02184575  02248663  02274892 | Oxytetracycline (as Dihydrate) | For the treatment of bacterial pneumonia, pasteurellosis (associated with shipping fever complex), mastitis, metritis, calf scours (bacterial enteritis), foot rot, navel ill, calf diphtheria, leptospirosis, blackleg/malignant edema, peritonitis, joint ill. | Administer by deep intramuscular injection or subcutaneous injection at a single dose rate of 20 mg oxytetracycline per kg bodyweight. The injectable solution is specially formulated to provide sustained antibiotic blood levels **up to 4 days** in cattle following a single treatment. | Do not use in lactating dairy cattle. | The course dosage is **20** mg/kg/course. The duration of action is 4 days. The daily dosage is 20/4 = **5** mg/kg/day. | **5.0** | **20** | 5*300 = **1,500** | 20*300 = **6,000** | **QJ01AA06** (Antibacterials for systemic use – tetracyclines) |
| 02182890  02248742 | Oxytetracycline (as Dihydrate) | For the treatment of bacterial pneumonia, pasteurellosis (associated with the shipping fever complex) and foot rot caused or complicated by bacteria susceptible to oxytetracycline, and for the treatment of pinkeye (IBK) caused by *Moraxella bovis*. | Designed for intramuscular (02182890 and 02248742) or subcutaneous (02182890) administration at the single dose rate of 20 mg oxytetracycline per kg body weight. It provides sustained antibiotic blood level action **over a 3 to 5 day period** in cattle following a single treatment. | Do not use in lactating dairy cattle. | The course dosage is **20** mg/kg/course. The duration of action is 4 days. The daily dosage is 20/4 = **5** mg/kg/day. | **5.0** | **20** | 5*300 = **1,500** | 20*300 = **6,000** | **QJ01AA06** (Antibacterials for systemic use – tetracyclines) |
| 02157209 | Oxytetracycline (as Dihydrate) | For the treatment of bacterial pneumonia, pasteurellosis (associated with the shipping fever complex) and foot rot caused or complicated by bacteria susceptible to oxytetracycline, and for the treatment of pinkeye (IBK) caused by *Moraxella bovis*. | Designed for intramuscular or subcutaneous administration at the single dose rate of 20 mg oxytetracycline per kg body weight. It provides sustained antibiotic blood level action **over a period of days** in the intended species following a single treatment. | Do not use in lactating dairy cattle. | The course dosage is **20** mg/kg/course. The duration of action is unknown. | Not determined | **20** | Not determined | 20*300 = **6,000** | **QJ01AA06** (Antibacterials for systemic use – tetracyclines) |
| 02387719 | Tildipirosin | For the **treatment of BRD** associated with *Mannheimia haemolytica*, *Pasteurella multocida* and *Histophilus somni*. For the **reduction of morbidity associated with BRD** in feedlot calves, caused by *Mannheimia haemolytica*, *Pasteurella multocida* and *Histophilus somni*, during the first 14 days in the feedlot, when administered at the time of arrival. | Should be administered in the neck **by a single subcutaneous injection** at a dose rate of 4 mg/kg body weight. Most animals will respond to treatment within 3 to 5 days. | Not for use in breeding cattle. Do not use in female dairy cattle 20 months of age or older. | The course dosage is **4** mg/kg/course. The daily dosage is defined as 4/7 ≈ **0.57** mg/kg/day. | **0.57** | **4.0** | 4*300/7 ≈ **171** | 4*300 = **1,200** | **QJ01FA96** (Antibacterials for systemic use – macrolides, lincosamides and streptogramins) |
| 02431912 | Tilmicosin | For the **treatment of BRD** associated with *Mannheimia haemolytica* and *Pasteurella multocida*. | Administer a **single subcutaneous injection of 10 mg tilmicosin/kg body weight**. If no improvement is noted within 48 hours, the diagnosis should be reconfirmed. | Do not use in lactating dairy cattle. | The course dosage is **10** mg/kg/course. The daily dosage is defined as 10/7 ≈ **1.4** mg/kg/day. | **1.4** | **10** | 10*300/7 ≈ **429** | 10*300 = **3,000** | **QJ01FA91** (Antibacterials for systemic use – macrolides, lincosamides and streptogramins) |
| 00857602  02435861 | Tilmicosin | For the **treatment of BRD** associated with *Mannheimia haemolytica* and *Pasteurella multocida*. For the **reduction of morbidity associated with BRD** in feedlot calves, caused by *Mannheimia haemolytica* and *Pasteurella multocida*, during the first 30 days in the feedlot, when administered at the time of arrival. |  |  |  |  |  |  |  |  |
| 02285452 | Tulathromycin | For the **treatment of BRD** associated with *Mannheimia haemolytica*, *Pasteurella multocida*, *Histophilus somni* and *Mycoplasma bovis*. For the **reduction of morbidity associated with BRD** in feedlot calves caused by *Mannheimia haemolytica*, *Pasteurella multocida*, *Histophilus somni* and *Mycoplasma bovis*, during the first 14 days in the feedlot when administered at the time of arrival. For the **treatment of IBK** associated with *Moraxella bovis*. For the **treatment of bovine foot rot** (interdigital necrobacillosis) associated with *Fusobacterium necrophorum* and *Porphyromonas levii*. | Inject subcutaneously in the neck, **a single dose** of 2.5 mg/kg body weight. Most animals will respond to treatment within 3 to 5 days. | Do not use in dairy cows 20 months of age or older. | The course dosage is **2.5** mg/kg/course. The daily dosage is defined as 2.5/7 ≈ **0.36** mg/kg/day. | **0.36** | **2.5** | 2.5*300/7 ≈ **107** | 2.5*300 = **750** | **QJ01FA94** (Antibacterials for systemic use – macrolides, lincosamides and streptogramins) |
| 00103594 | Tylosin | Beef cattle: For the treatment of pneumonia caused by strains of *Pasteurella multocida* and *Actinomyces pyogenes* susceptible to tylosin and contagious calf pneumonia and metritis caused by *Actinomyces pyogenes*. | Inject intramuscularly a **daily dose** of 17.6 mg of tylosin per kg body weight (8 mg per pound body weight). Do not exceed 5 days of treatment. If no improvement is observed in 3 days, the diagnosis should be reconfirmed. | Do not use in lactating dairy cattle. | The daily dosage is **17.6** mg/kg/day. The average duration of treatment is suspected to be 4 days (3 to 5 days). The course dosage is 17.6*4 = **70.4** mg/kg/course. | **17.6** | **70.4** | 17.6*300 = **5,280** | 70.4*300 = **21,120** | **QJ01FA90** (Antibacterials for systemic use – macrolides, lincosamides and streptogramins) |

**Supplementary 2.3. Average daily dosage, course dosage, daily dose, and course dose, for procaine benzylpenicillin (not combined), ceftiofur, and oxytetracycline (from injectable products).**

1. **Procaine Benzylpenicillin (not combined with Benzathine Benzylpenicillin)**

Three daily dosages have been identified: 12.6, 7.2, and 6.7 mg/kg/day, for an average daily dosage of **8.8 mg/kg/day**. Two course dosages have been identified: 50.4 and 30.0 mg/kg/course, for an average course dosage of **40.2 mg/kg/course**. Three daily doses have been calculated: 8.19, 4.68, and 2.01 g/animal/day, for an average daily dose of **4.96 g/animal/day**. Two course doses have been calculated: 32.76 and 9.0, for an average course dose of **20.88 g/animal/course**.

1. **Ceftiofur**

Three daily dosages have been identified: 1.0, 1.6, and 1.1 mg/kg/day, for an average daily dosage of **1.2 mg/kg/day**. Three course dosages have been identified: 4.0, 7.5, and 6.6 mg/kg/course, for an average course dosage of **6.0 mg/kg/course**. Three daily doses have been calculated: 0.65, 1.04, and 0.715 g/animal/day, for an average daily dose of **0.80 g/animal/day**. Three course doses have been calculated: 2.6, 4.875, and 4.29 g/animal/course, for an average course dose of **3.92 g/animal/course**.

1. **Oxytetracycline**

Two daily dosages have been identified: 6.7 and 5.0 mg/kg/day, for an average daily dosage of **5.9 mg/kg/day**. Two course dosages have been identified: 16.7 and 20 mg/kg/course, for an average course dosage of **18.4 mg/kg/course**. Three daily doses have been calculated: 4.355, 2.01, and 1.5 g/animal/day, for an average daily dose of **2.62 g/animal/day**. Three course doses have been calculated: 10.855, 5.01, and 6.0 g/animal/course, for an average course dose of **7.29 g/animal/course**.

**Supplementary 3.1. Details of the 27 oral products other than medicated premixes, containing exactly one antimicrobial agent, and marketed in Canada with at least one indication for cattle.**

Legend: **ATCvet**: Anatomical Therapeutic Chemical Classification System for veterinary medicinal products; **BRD**: Bovine Respiratory Diseases; **DIN**: Drug Identification Number.

A level tablespoon and a level teaspoon were assumed to contain 9 and 3 grams of soluble powder respectively. A rounded tablespoon and a rounded teaspoon were assumed to contain one third more than the level spoon (12 and 4 grams of soluble powder respectively). These approximations were used only when no other indication was given in the label. For suspensions and solutions, a tablespoon and a teaspoon were assumed to contain 15 and 5 milliliters respectively. For products with a dose for calves under 45 kg and a dose for calves over 45 kg (tetracycline), an average of both doses was computed and assigned to a body weight of 45 kg for calculations.

| **DIN of products marketed in Canada** | **Antimicrobial agent** | **Formulation** | **Type of cattle targeted by the product** | **Product Monograph** | **Concentration in the product** | **Calculations from the product monograph** | **Daily dosage (mg/kg/day)** | **Course dosage (mg/kg/course)** | **Daily dose (mg/animal/day)** | **Course dose (mg/animal/course)** | **ATCvet Code** |
| --- | --- | --- | --- | --- | --- | --- | --- | --- | --- | --- | --- |
| 02451999 | Monensin (as sodium) | Controlled release capsule | Lactating dairy cattle  (**650 kg**) | **1-** As an aid in the prevention of ketosis and subclinical ketosis (hyperketonemia) in lactating dairy cattle. **2-** For the reduction in fecal shedding of *Mycobacterium avium paratuberculosis* in mature dairy cattle in high risk Johne’s disease herds as an aid in the herd control of Johne’s disease as one component of a multi-component Johne’s disease control program. The capsule delivers an approximate average dose of 335 mg of monensin per day for approximately 95 days. For indications 1 and 2, administer one capsule orally 2 to 4 weeks prior to expected calving date. | 32.4 g of monensin per capsule | The course dose is **32400** mg/animal/course. The daily dose is **335** mg/animal/day.  The course dosage is 32400/650 ≈ **49.8** mg/kg/course. The daily dosage is 335/650 ≈ **0.52** mg/kg/day. | **0.52** | **49.8** | **335** | **32,400** | **QA16QA06** (other alimentary tract and metabolism products for veterinary use – drugs for prevention and/or treatment of acetonemia) |
| 00527750  01981390  02438607 | Neomycin Sulfate | Water Soluble Powder | Calves  (**100 kg**) | As an aid in the treatment of bacterial enteritis. Individual Treatment: Add to the drinking water consumed daily, 1 g of powder per 50 kg of body weight. Do not administer medicated drinking water for more than 3 days in a row at this dosage rate. | 812.5 mg of neomycin sulfate per gram of powder | The daily dosage is 812.5/50 = **16.25** mg/kg/day. The course dosage is 16.25*3 = **48.75** mg/kg/course. | **16.3** | **48.8** | 16.3*100 = **1,630** | 48.8*100 = **4,880** | **QA07AA01** (Intestinal antiinfectives – antibiotics) |
| 00560189  02438593 | Oxytetracycline | Water Soluble Powder | Calves  (**100 kg**) | **1-** As an aid in the treatment of enteritis. Add 1000 mg of oxytetracycline hydrochloride per 200 kg of body weight every 12 hours, given as a drench in water, for 3 to 5 days. **2-** As an aid in the treatment of respiratory diseases. Double the above dosage for 3 to 5 days. | 62.5 mg (00560189) and 1000 mg (02438593) of oxytetracycline hydrochloride per gram of powder | Interpretation: The average duration of treatment is 4 days. The average dose is 3000 mg (2000 or 4000) of oxytetracycline per 200 kg per day.  The average daily dosage is 3000/200 = **15** mg/kg/day. The average course dosage is 15*4 = **60** mg/kg/course. | **15** | **60** | 15*100 = **1,500** | 60*100 = **6,000** | **QJ01AA06** (Antibacterials for systemic use – tetracyclines) |
| 02256983 | Oxytetracycline | Water Soluble Powder | Calves  (**100 kg**) | As an aid in the treatment of bacterial scours and bacterial pneumonia. For individual treatment, 5 g per 1000 kg body weight dissolved in water, twice daily for 2 to 3 days. | 1000 mg of oxytetracycline hydrochloride per gram of powder | The daily dosage is 5*1000*2/1000 = **10** mg/kg/day. The course dosage is 10*2.5 = **25** mg/kg/course. | **10** | **25** | 10*100 = **1,000** | 25*100 = **2,500** | **QJ01AA06** (Antibacterials for systemic use – tetracyclines) |
| 00546704 | Oxytetracycline | Water Soluble Powder | All cattle including lactating dairy animals  (**300 kg**) | **1-** For treatment or prevention of scours. For treatment, use 4 grams per 45 kg in 60 to 90 mL of water or milk as a drench. Treat for 4 to 5 days. **2-** For treatment or prevention of respiratory diseases. For treatment or prevention, use 4 to 8 grams per 45 kg of body weight in drinking water or in 60 to 90 mL water or milk as a drench.  Interpretation: For respiratory diseases, the treatment dose is 8 grams per 45 kg of body weight per day (the prevention dose is 4 grams). | 55 mg of oxytetracycline hydrochloride per gram of powder | Interpretation: The average duration of treatment is 4.5 days. The average dose is 6 grams per 45 kg per day.  The average daily dosage is 6*55/45 ≈ **7.3** mg/kg/day. The average course dosage is 6*55*4.5/45 = **33** mg/kg/course. | **7.3** | **33** | 7.3*300 = **2,190** | 33*300 = **9,900** | **QJ01AA06** (Antibacterials for systemic use – tetracyclines) |
| 00546712 | Oxytetracycline | Water Soluble Powder | All cattle including lactating dairy animals  (**300 kg**) | **1-** As an aid in the prevention or treatment of scours. For treatment, use 0.5 teaspoon per 45 kg in 60 to 90 mL water or milk as a drench. Treat for 4 to 5 days. **2-** As an aid in the prevention or treatment of respiratory diseases. For treatment or prevention, use 0.5 to 1 teaspoon per 45 kg of bodyweight in drinking water or in 60 to 90 mL water or milk as a drench.  Interpretation: For respiratory diseases, the treatment dose is 1 teaspoon per 45 kg of body weight per day (the prevention dose is 0.5 teaspoon). | 220 mg of oxytetracycline hydrochloride per gram of powder (**1000 mg per teaspoon**) | Interpretation: The average duration of treatment is 4.5 days. The average dose is 0.75 teaspoon per 45 kg per day.  The average daily dosage is 0.75*1000/45 ≈ **16.7** mg/kg/day. The average course dosage is 0.75*1000*4.5/45 = **75** mg/kg/course. | **16.7** | **75** | 16.7*300 = **5,010** | 75*300 = **22,500** | **QJ01AA06** (Antibacterials for systemic use – tetracyclines) |
| 01908375 | Sulfamethazine | Sustained release tablets | Do not use in lactating dairy cattle.  **(300 kg)** | For the treatment of infectious diseases including colibacillosis, bacterial pneumonia, calf diphtheria, bovine respiratory disease complex (shipping fever complex), and necrotic pododermatitis (foot rot). One tablet for each 20 kg of body weight. A single dose will provide adequate blood sulfonamide levels that will be sustained for at least 4 days. | 8 grams of sulfamethazine per tablet | Interpretation: The average duration of action is 4 days.  The course dosage is 8000/20 = **400** mg/kg/course. The daily dosage is 400/4 = **100** mg/kg/day. | **100** | **400** | 100*300 = **30,000** | 400*300 = **120,000** | **QJ01EQ03** (Antibacterials for systemic use – sulfonamides) |
| 02224194 | Sulfamethazine | Sustained release boluses | Do not administer to cattle weighing less than 136 kg and to lactating dairy cattle.  **(300 kg)** | For the treatment of bacterial pneumonia and bovine respiratory disease complex (shipping fever complex), necrotic pododermatitis (foot rot), and acute metritis. Administer at a dose rate of one bolus for each 91 kg body weight. Boluses are designed to provide a therapeutic sulfamethazine level in approximately 6 hours, and persist in providing this level for 72 hours. In more severe cases, it is strongly recommended that a second dose be given to provide an additional 72 hours of therapy. For the second administration use the same dose schedule as above. | 32.1 grams of sulfamethazine per bolus | Interpretation: The average duration of action is 3 days after one administration. The course dose is either 1 bolus or 2 boluses (average 1.5 bolus) per 91 kg of body weight.  The average course dosage is 32100*1.5/91 ≈ **529.12** mg/kg/course. The daily dosage is 32100/(3*91) ≈ **117.58** mg/kg/day. | **117.6** | **529.1** | 117.6*300 = **35,280** | 529.1*300 = **158,730** | **QJ01EQ03** (Antibacterials for systemic use – sulfonamides) |
| 00251828  00308382 | Sulfamethazine | Boluses | All cattle including lactating dairy animals  (**300 kg**) | As an aid in the treatment of bacterial pneumonia, metritis, foot rot, diphtheria, and other conditions in food animals, when caused by sulfamethazine susceptible microorganisms. Initial Dose: 1 bolus per 80 kg bodyweight. Second Dose: 0.5 bolus per 80 kg bodyweight, 12 hours later if necessary. Caution: If no improvement is noted within 4 days, the diagnosis should be re-evaluated. | 15 grams of sulfamethazine per bolus | Interpretation: The course dose is either 1 bolus or 1.5 bolus, for an average of 1.25 bolus per 80 kg of body weight. The duration of action is unknown.  The average course dosage is 1.25*15000/80 = **234.375** mg/kg/course. | **Not determined** | **234.4** | **Not determined** | 234.4*300 = **70,320** | **QJ01EQ03** (Antibacterials for systemic use – sulfonamides) |
| 00097187 | Sulfamethazine | Oral solutions | All cattle including lactating dairy animals  (**300 kg**) | For the treatment of bacterial infections such as metritis, bacterial enteritis (scours), acute mastitis, acute bacterial respiratory infections. Cattle, calves: Give 2.5 mL/45 kg bodyweight for the first day, then reduce the dose by ½ for the next 3 days. Beef cattle: Mix 60 mL/4.5 L water for the first day, then reduce the dose by ½ for the next 3 days. Medicated water should be the only source of water during treatment.  Approximation used for calculations:  For beef cattle, the concentration in water is 1666.7 mg of sulfamethazine per liter the first day, and 833.3 mg/L the 3 following days. A daily water intake of 10% is used. | 125 mg of sulfamethazine sodium per mL | Cattle, calves: The course dosage is (2.5*125+1.25*125*3)/45 ≈ **17.36** mg/kg/course. The daily dosage is 17.36/4 ≈ **4.34** mg/kg/day.  Beef cattle: The course dosage is (1666.7+3*833.3)*10% = **416.66** mg/kg/course. The daily dosage is 416.66/4 ≈ **104.17** mg/kg/day.  The average course dosage is (17.36+416.66)/2 ≈ 217.0 mg/kg/course. The average daily dosage is (4.34+104.17)/2 ≈ 54.3 mg/kg/day. | **54.3** | **217.0** | 54.3*300 = **16,290** | 217.0*300 = **65,100** | **QJ01EQ03** (Antibacterials for systemic use – sulfonamides) |
| 00097195  00308218 | Sulfamethazine | Oral solutions | All cattle including lactating dairy animals  (**300 kg**) | For the treatment of bacterial infections such as metritis, bacterial enteritis (scours), acute mastitis, acute bacterial respiratory infections. Cattle, calves: Give 25 mL/40 kg bodyweight for the first day, then reduce the dose by ½ for the next 3 days. Beef cattle: Mix 25 mL/4 L water for the first day, then reduce the dose by ½ for the next 3 days. Medicated water should be the only source of water during treatment.  Approximation used for calculations:  For beef cattle, the concentration in water is 1562.5 mg of sulfamethazine per liter the first day, and 781.25 mg/L the 3 following days. A daily water intake of 10% is used. | 250 mg of sulfamethazine sodium per mL | Cattle, calves: The course dosage is (25*250+12.5*250*3)/40 ≈ **390.6** mg/kg/course. The daily dosage is 390.6/4 ≈ **97.7** mg/kg/day.  Beef cattle: The course dosage is (1562.5+3*781.25)*10% ≈ **390.6** mg/kg/course. The daily dosage is 390.6/4 ≈ **97.7** mg/kg/day.  The average course dosage is **390.6** mg/kg/course. The average daily dosage is **97.7** mg/kg/day. | **97.7** | **390.6** | 97.7*300 = **29,310** | 390.6*300 = **117,180** | **QJ01EQ03** (Antibacterials for systemic use – sulfonamides) |
| 00601837  02391368 | Sulfamethazine | Oral solutions | All cattle including lactating dairy animals  (**300 kg**) | Cattle: As an aid in the treatment of foot rot, shipping fever, bacterial enteritis/scours, metritis, mastitis and bacterial respiratory diseases. Calves: As an aid in the treatment of bacterial respiratory infections and secondary infections in bacterial enteritis/scours. As an aid in the prevention of coccidiosis.  Dairy cattle, calves: First day: 45 mL for each 50 kg of body weight. Following days: one half of the above dosage. Do not treat for more than 5 days.  Beef cattle: First day: for each 100 kg of body weight, add 100 mL into the amount of water that will be consumed daily by the animals. Following days: one half of the above dosage. Do not treat for more than 5 days. | 250 mg of sulfamethazine sodium per mL | Dairy cattle, calves: The course dosage is (45*250+22.5*250*4)/50 = **675** mg/kg/course. The daily dosage is 675/5 = **135** mg/kg/day.  Beef cattle: The course dosage is (100*250+50*250*4)/100 = **750** mg/kg/course. The daily dosage is 750/5 = **150** mg/kg/day.  The average daily dosage is (135+150)/2 = **142.5** mg/kg/day. The average course dosage is (675+750)/2 = **712.5** mg/kg/course. | **142.5** | **712.5** | 142.5*300 = **42,750** | 712.5*300 = **213,750** | **QJ01EQ03** (Antibacterials for systemic use – sulfonamides) |
| 00293172  00308358 | Sulfamethazine | Water Soluble Powder | All beef cattle  (**300 kg**) | For the treatment of bacterial infections such as metritis, bacterial enteritis, and acute respiratory infections. No individual dosage. First day: Mix 1 kg of powder in 640 L of drinking water. Second, third, and fourth days: Mix 1 kg of powder in 1280 L of drinking water.  Approximation used for calculations: The concentration in water is 1562.5 mg of sulfamethazine per liter the first day, and 781.25 mg per liter the 3 following days. A daily water intake of 10% is used. | 1000 mg of sulfamethazine sodium per gram of powder | The course dosage is (1562.5+3*781.25)*10% = **390.625** mg/kg/course. The daily dosage is 390.625/4 ≈ **97.7** mg/kg/day. | **97.7** | **390.6** | 97.7*300 = **29,310** | 390.6*300 = **117,180** | **QJ01EQ03** (Antibacterials for systemic use – sulfonamides) |
| 00319414 | Sulfapyridine | Boluses | All cattle including lactating dairy animals  (**300 kg**) | As an aid in the treatment of foot rot in cattle when caused by *Sphaerophorus necrophorus* and/or other organisms susceptible to sulfapyridine or organic iodide. Initially, administer orally 1 to 1.5 boluses for each 200 pounds of body weight. Repeat every 12 hours with 1 bolus for each 400 pounds of body weight for 2 to 3 days. | 13 g of sulfapyridine per bolus | Interpretation: The average duration of treatment is 3 days. Day 1: 1.25 boluses per 90.7 kg + 1 bolus per 181.4 kg. Day 2: 2 boluses per 181.4 kg. Day 3: 2 boluses per 181.4 kg.  The average course dosage is (1.25*13/90.7+5*13/181.4)*1000 ≈ **537.49** mg/kg/course. The average daily dosage is 537.49/3 ≈ **179.16** mg/kg/day. | **179.2** | **537.5** | 179.2*300 = **53,760** | 537.5*300 = **161,250** | **QJ01EQ04** (Antibacterials for systemic use – sulfonamides) |
| 00283851  00308153 | Tetracycline | Water Soluble Powder | Calves  (**100 kg**) | For the treatment of infections caused by microorganisms susceptible to tetracycline. Calves: For bacterial scours, give 1 level teaspoon once daily (for calves under 45 kg) or 2 level teaspoons once daily (for calves over 45 kg). | 55 mg of tetracycline hydrochloride per gram of powder (**175 mg per teaspoon**) | Approximation used for calculations: 1.5 level teaspoon per 45 kg per day. The daily dosage is 1.5*175/45 ≈ **5.8** mg/kg/day. The duration of treatment is unknown. | **5.8** | **Not determined** | 5.8*100 = **580** | **Not determined** | **QJ01AA07** (Antibacterials for systemic use – tetracyclines) |
| 00526614  00530646  00780863  00894486 | Tetracycline | Water Soluble Powder | Calves  (**100 kg**) | **1-** As an aid in the treatment of scours. Add in water 250 mg of tetracycline hydrochloride per 50 kg of body weight every 12 hours, given as a drench, for 3 to 5 days. **2-** As an aid in the treatment of respiratory diseases. Double the above dosage for 3 to 5 days. | 250 mg (00526614, 00780863) and 1000 mg (00530646, 00894486) of tetracycline hydrochloride per gram of powder | Interpretation: The average duration of treatment is 4 days. The average dose is 750 mg (500 or 1000 mg) of tetracycline per 50 kg per day.  The average daily dosage is 750/50 = **15** mg/kg/day. The average course dosage is 15*4 = **60** mg/kg/course. | **15** | **60** | 15*100 = **1,500** | 60*100 = **6,000** | **QJ01AA07** (Antibacterials for systemic use – tetracyclines) |

**Supplementary 3.2. Average daily dosage, course dosage, daily dose, and course dose, for oxytetracycline, sulfamethazine (sulfadimidine), and tetracycline (from oral products other than medicated premixes, containing exactly one antimicrobial agent).**

1. **Oxytetracycline**

Water soluble powders: Four daily dosages have been identified (15, 10, 7.3, and 16.7), for an average daily dosage of **12.3 mg/kg/day**. Four course dosages have been identified (60, 25, 33, and 75), for an average course dosage of **48.3 mg/kg/course**. Four daily doses have been calculated (1.5, 1.0, 2.19, and 5.01), for an average daily dose of **2.43 g/animal/day**. Four course doses have been calculated (6.0, 2.5, 9.9, and 22.5), for an average course dose of **10.23 g/animal/course**.

1. **Sulfamethazine (Sulfadimidine)**

Boluses: Two daily dosages have been identified (100 and 117.6), for an average daily dosage of **108.8 mg/kg/day**. Three course dosages have been identified (400, 529.1, and 234.4), for an average course dosage of **387.8 mg/kg/course**. Two daily doses have been calculated (30 and 35.28), for an average daily dose of **32.64 g/animal/day**. Three course doses have been calculated (120, 158.73, and 70.32), for an average course dose of **116.35 g/animal/course**.

Oral solutions: Three daily dosages have been identified (54.3, 97.7, and 142.5), for an average daily dosage of **98.2 mg/kg/day**. Three course dosages have been identified (217.0, 390.6, and 712.5), for an average course dosage of **440.0 mg/kg/course**. Three daily doses have been calculated (16.29, 29.31, and 42.75), for an average daily dose of **29.45 g/animal/day**. Three course doses have been calculated (65.1, 117.18, and 213.75), for an average course dose of **132.01 g/animal/course**.

Water soluble powders: One daily dosage has been identified: **97.7 mg/kg/day**. One course dosage has been identified: **390.6 mg/kg/course**. One daily dose has been calculated: **29.31 g/animal/day**. One course dose has been calculated: **117.18 g/animal/course**.

Average values (boluses, solutions, water soluble powders): The average daily dosage is (108.8+98.2+97.7)/3 ≈ **101.6 mg/kg/day**. The average course dosage is (387.8+440.0+390.6)/3 ≈ **406.1 mg/kg/course**. The average daily dose is (32.64+29.45+29.31)/3 ≈ **30.47 g/animal/day**. The average course dose is (116.35+132.01+117.18)/3 ≈ **121.85 g/animal/course**.

1. **Tetracycline**

Water soluble powders: Two daily dosages have been identified (5.8 and 15), for an average daily dosage of **10.4 mg/kg/day**. One course dosage has been identified: **60 mg/kg/course**. Two daily doses have been calculated (0.58 and 1.5), for an average daily dose of **1.04 g/animal/day**. One course dose has been calculated: **6.0 g/animal/course**.

**Supplementary 3.3. Details of the 23 medicated premixes containing exactly one antimicrobial agent, and marketed in Canada with at least one indication for cattle.**

Legend: **ATCvet**: Anatomical Therapeutic Chemical Classification System for veterinary medicinal products; **DIN**: Drug Identification Number.

When the period of exposition is not precisely indicated, a period of exposition of **3 months (or 3*30 = 90 days)** is used for calculation of a course dosage / course dose. The dry matter intake is set at **2% (0.02)** for calculations.

| **DIN of products marketed in Canada** | **Antimicrobial agent** | **Product Monograph** | **Concentration in the product** | **Calculations from the product monograph** | **Daily dosage (mg/kg/day)** | **Course dosage (mg/kg/course)** | **Daily dose (mg/animal/day)** | **Course dose (mg/animal/course)** | **ATCvet Code** |
| --- | --- | --- | --- | --- | --- | --- | --- | --- | --- |
| 00641804  00698210  02407558 | Chlortetracycline (Category III) | **● As an aid in the prevention of foot rot in beef cattle and non-lactating dairy cattle.** Level in the feed such that each animal will receive 0.22 mg/kg of body weight per day or 70 mg/head/day. Feed continuously during the period animals are exposed to this disease.  **● As an aid in the prevention of bacterial diarrhea in calves up to 136 kg.** 55 mg/kg of complete feed (or of milk replacer powder). Feed continuously during periods of early growth. | **110 g** (DIN 00641804) and **220 g** (DIN 00698210 and 02407558) of chlortetracycline per kg | **● Beef cattle and non-lactating dairy cattle.**  Daily dosage: **0.22** mg/kg/day  Course dosage: 0.22*90 = **19.8** mg/kg/course  Daily dose: **70** mg/animal/day  Course dose: 70*90 = **6,300** mg/animal/course  **● Calves up to 136 kg (weight used: 100 kg).**  Daily dosage: 55*0.02 = **1.1** mg/kg/day  Course dosage: 1.1*90 = **99** mg/kg/course  Daily dose: 1.1*100 = **110** mg/animal/day  Course dose: 99*100 = **9,900** mg/animal/course | (0.22+1.1)/2 = **0.66** | (19.8+99)/2 = **59.4** | (70+110)/2 = **90** | (6300+9900)/2 = **8,100** | **QJ01AA03** (Antibacterials for systemic use – tetracyclines) |
| 02321920  02321939 | Lasalocid (Category IV) | **● For improved feed efficiency and increased rate of weight gain in feedlot cattle being fed in confinement for slaughter.** Level of drug in complete diet: 36 mg of lasalocid sodium per kg of diet (100% dry matter basis). Level of drug in supplements: 250 mg/head/day for cattle weighing 200-299 kg, and 350 mg/head/day for cattle weighing 300 kg or greater. Feed continuously until market weight.  **● For increased rate of weight gain in pasture cattle (stocker, feeder cattle, and beef and dairy replacement heifers).** 200 mg of lasalocid sodium/animal/day from the beginning to end of the pasture season.  **● As an aid in the prevention of coccidiosis caused by *Eimeria bovis* and *Eimeria zuernii* in calves up to 360 kg of body weight being fed in confinement.** Level of drug in complete diet: 36 mg of lasalocid sodium per kg of diet (100% dry matter basis). Level of drug in supplements: 100 mg/head/day for cattle weighing 50-99 kg, 150 for 100-149 kg, 200 for cattle 150-199 kg, 250 for cattle 200-249 kg, 300 for cattle 250-299 kg, 350 for cattle 300-360 kg. Feed continuously during periods of exposure to coccidiosis or when coccidiosis is likely to be a hazard. | **200 g** of lasalocid sodium activity per kg | **● Feedlot cattle (weight used: 300 kg).**  From mixing directions in complete diet:  Daily dosage: 36*0.02 = **0.72** mg/kg/day  Course dosage: 0.72*90 = **64.8** mg/kg/course  Daily dose: 0.72*300 = **216** mg/animal/course  Course dose: 216*90 = **19,440** mg/animal/course  From mixing directions in supplements:  Daily dosage: **1** mg/kg/day  Course dosage: 1*90 = **90** mg/kg/course  Daily dose: 1*300 = **300** mg/animal/day  Course dose: 90*300 = **27,000** mg/animal/course  **● Pasture cattle (weight used: 300 kg).**  Daily dosage: 200/300 ≈ **0.67** mg/kg/day  Course dosage: 90*200/300 = **60** mg/kg/course  Daily dose: **200** mg/animal/day  Course dose: 200*90 = **18,000** mg/animal/course  **● Calves up to 360 kg (weight used: 300 kg).**  From mixing directions in complete diet:  Daily dosage: 36*0.02 = **0.72** mg/kg/day  Course dosage: 0.72*90 = **64.8** mg/kg/course  Daily dose: 0.72*300 = **216** mg/animal/course  Course dose: 216*90 = **19,440** mg/animal/course  From mixing directions in supplements:  Daily dosage: (100/74.5+150/124.5+200/174.5+250/224.5+300/274.5+350/330)/6 ≈ **1.16** mg/kg/day  Course dosage: 1.16*90 =**104.4** mg/kg/course  Daily dose: 1.16*300 =**348** mg/animal/day  Course dose: 348*90 = **31,320** mg/animal/course | (0.72+1+0.67+1.16)/4 ≈ **0.89** | (64.8+90+60+104.4)/4 ≈ **79.8** | (216+300+200+348)/4 = **266** | (19440+27000+18000+31320)/4 ≈ **23,940** | **QP51AH02** (Agents against protozoal diseases – pyranes and hydropyranes) |
| 02231172  02231173  02243232 | Monensin (Category IV) | **● Claim 1.** For improved feed efficiency in beef cattle (steers and heifers) fed in confinement for slaughter. Feed continuously at a rate of not less than 33 g and not more than 48 g monensin activity per tonne until animals reach market weight.  **● Claim 2.** As an aid in the prevention of coccidiosis caused by *Eimeria bovis* and *Eimeria zuernii* in cattle. Feed continuously at a rate of 22 g monensin activity per tonne of feed during periods of exposure to coccidiosis or when coccidiosis is likely to be a hazard. Note to user: Coccidiosis occurs sporadically in first lactation dairy heifers, but is not considered a significant disease in mature dairy cows.  **● Claim 3.** For increased rate of weight gain in growing cattle on pasture (slaughter, stocker and feeder cattle, and beef and dairy replacement heifers) of greater than 180 kg body weight. Hand feed at 200 mg of monensin activity per head per day in medicated supplement.  **● Claim 4.** For reduction of milk fat percentage in dairy cows. Feed continuously at a rate of not less than 16 g and not more than 24 g monensin activity per tonne.  **● Claim 5.** For minimizing loss of body condition during lactation in dairy cows. Feed continuously at a rate of not less than 8 g and not more than 24 g monensin activity per tonne.  **● Claim 6.** For improving feed efficiency of milk protein production in dairy cows. Feed continuously at a rate of not less than 16 g and not more than 24 g monensin activity per tonne. | **200 g** of monensin sodium activity per kg | **● Claim 1 (weight used: 300 kg).**  Daily dosage: (33+48)*0.02/2 = **0.81** mg/kg/day  Course dosage: 0.81*90 = **72.9** mg/kg/course  Daily dose: 0.81*300 = **243** mg/animal/day  Course dose: 72.9*300 = **21,870** mg/animal/course  **● Claim 2 (weight used: 300 kg).**  Daily dosage: 22*0.02 = **0.44** mg/kg/day  Course dosage: 0.44*90 = **39.6** mg/kg/course  Daily dose: 0.44*300 = **132** mg/animal/day  Course dose: 39.6*300 = **11,880** mg/animal/course  **● Claim 3 (weight used: 300 kg).**  Daily dosage: 200/300 ≈ **0.67** mg/kg/day  Course dosage: 90*200/300 = **60** mg/kg/course  Daily dose: **200** mg/animal/day  Course dose: 200*90 = **18,000** mg/animal/course  **● Claim 4 and 6 (weight used: 650 kg).**  Daily dosage: (16+24)*0.02/2 = **0.4** mg/kg/day  Course dosage: 0.4*90 = **36** mg/kg/course  Daily dose: 0.4*650 = **260** mg/animal/day  Course dose: 36*300 = **23,400** mg/animal/course  **● Claim 5 (weight used: 650 kg).**  Daily dosage: (8+24)*0.02/2 = **0.32** mg/kg/day  Course dosage: 0.32*90 = **28.8** mg/kg/course  Daily dose: 0.32*650 = **208** mg/animal/day  Course dose: 28.8*650 = **18,720** mg/animal/course | (0.81+0.44+0.67+0.4+0.32)/5 ≈ **0.53** | (72.9+39.6+60+36+28.8)/5 ≈ **47.5** | (243+132+200+260+208)/5 = **208.6** | (21870+11880+18000+23400+18720)/5 = **18,774** | **QP51AH03** (Agents against protozoal diseases – pyranes and hydropyranes) |
| 00637513  00654787  00719315  02246806  02246807  02246808 | Oxytetracycline (Category III) | **● Beef cattle: As an aid in reducing the incidence of bloat in young cattle on pasture and in feedlots.** Feed continuously. Level of oxytetracycline hydrochloride in the complete feed is to be such that each animal will receive 75 mg per day. 55 g of oxytetracycline hydrochloride per tonne of complete feed when fed at the rate of 1.36 kg per head per day meets the 75 mg level stated above.  **● Calves up to 136 kg: As an aid in the prevention of bacterial enteritis.** Feed continuously during the period of early growth 55 mg of oxytetracycline hydrochloride per kg of complete feed. | **110 g** (DIN 00637513 and 02246806), **220 g** (DIN 00654787 and 02246807), and **440 g** (DIN 00719315 and 02246808) of oxytetracycline per kg | **● Beef cattle.**  Daily dosage: 55*0.02 = **1.1** mg/kg/day  Course dosage: 1.1*90 = **99** mg/kg/course  Daily dose: **75** mg/animal/day  Course dose: 75*90 = **6,750** mg/animal/course  **● Calves up to 136 kg (weight used: 100 kg).**  Daily dosage: 55*0.02 = **1.1** mg/kg/day  Course dosage: 1.1*90 = **99** mg/kg/course  Daily dose: 1.1*100 = **110** mg/animal/day  Course dose: 99*100 = **9,900** mg/animal/course | **1.1** | **99** | **92.5** | **8,325** | **QJ01AA06** (Antibacterials for systemic use – tetracyclines) |
| 00603724  00884545 | Salinomycin (Category IV) | **● Steers:** For the improvement of feed efficiency in steers fed in confinement for slaughter. Recommended dosage of 100 mg of salinomycin sodium per head per day. The medicated feed is to be fed continuously to steers during their entire confinement period.  **● Feedlot Heifers:** For improved growth rate and feed efficiency, and as an aid in suppression of estrus in heifers being fed for slaughter. Note: This claim only applies when salinomycin sodium is administered concurrently with melengestrol acetate at 0.40 mg/head/day. Feed the medicated feed containing melengestrol acetate and salinomycin sodium to beef heifers **220 kg and over** being fed for slaughter such that each animal receiving 0.40 mg melengestrol acetate and 100 mg salinomycin sodium per head per day. | **60 g** of salinomycin sodium per kg | **● Steers and feedlot heifers (weight used: 300 kg).**  Daily dosage: 100/300 ≈ **0.33** mg/kg/day  Course dosage: 0.33*90 = **30** mg/kg/course  Daily dose: **100** mg/animal/day  Course dose: 100*90 = **9,000** mg/animal/course | **0.33** | **30** | **0.1** | **9.0** | **QP51AH01** (Agents against protozoal diseases – pyranes and hydropyranes) |
| 02240124  02349507 | Tilmicosin (Category II) | **● For the reduction of bovine respiratory disease (BRD) morbidity associated with *Mannheimia haemolytica*, *Pasteurella multocida* and/or *Histophilus somni* in groups of feedlot beef cattle** experiencing an outbreak of BRD, when: 1) clinical BRD has been diagnosed in at least 10% of animals in the group to be treated; AND 2) treatment is initiated within the first 45 days of arrival in the feedlot; AND 3) medication is limited to one single period of 14 consecutive days of treatment. Feed at the appropriate concentration in medicated feed to target **12.5 mg/kg body weight/day for a 14-day period**. | **200 g** of tilmicosin per kg | **● Feedlot beef cattle (weight used: 300 kg).**  Daily dosage: **12.5** mg/kg/day  Course dosage: 12.5*14 = **175** mg/kg/course  Daily dose: 12.5*300 = **3,750** mg/animal/day  Course dose: 175*300 = **52,500** mg/animal/course | **12.5** | **175** | **3,750** | **52,500** | **QJ01FA91** (Antibacterials for systemic use – macrolides, lincosamides and streptogramins) |
| 02242023  00474444  00713481  02472058  00800422 | Tylosin (Category II) | **● To reduce the incidence of liver abscesses caused by *Trueperella pyogenes* and *Fusobacterium necrophorum* in beef cattle (steers and heifers) fed in confinement for slaughter.** Administer at the rate of 11 g tylosin activity per 1,000 kg of complete diet including roughage (100% dry matter basis). Feed continuously as the sole ration. | **22 g** (DIN 00713481), **88 g** (DIN 00474444 and 00800422), and **220 g** (DIN 02242023 and 02472058) of tylosin per kg | **● Beef cattle (weight used: 300 kg).**  Daily dosage: 11*0.02 = **0.22** mg/kg/day  Course dosage: 0.22*90 = **19.8** mg/kg/course  Daily dose: 0.22*300 = **66** mg/animal/day  Course dose: 19.8*300 = **5,940** mg/animal/course | **0.22** | **19.8** | **66** | **5,940** | **QJ01FA90** (Antibacterials for systemic use – macrolides, lincosamides and streptogramins) |

**Supplementary 4.1. Details of the 16 oral products other than medicated premixes, containing exactly two antimicrobial agents, and marketed in Canada with at least one indication for cattle.**

Legend: **ATCvet**: Anatomical Therapeutic Chemical Classification System for veterinary medicinal products; **BRD**: Bovine Respiratory Diseases; **DIN**: Drug Identification Number; **DMI**: dry matter intake; **IU**: International Units.

A level tablespoon and a level teaspoon were assumed to contain 9 and 3 grams of soluble powder respectively. A rounded tablespoon and a rounded teaspoon were assumed to contain one third more than the level spoon (12 and 4 grams of soluble powder respectively). These approximations were used only when no other indication was given in the label. For suspensions and solutions, a tablespoon and a teaspoon were assumed to contain 15 and 5 milliliters respectively.

| **DIN of products marketed in Canada** | **Antimicrobial agent** | **Formulation** | **Type of cattle targeted by the product** | **Product Monograph** | **Concentration in the product** | **Calculations from the product monograph** | **Daily dosage (mg/kg/day)** | **Course dosage (mg/kg/course)** | **Daily dose (mg/animal/day)** | **Course dose (mg/animal/course)** | **ATCvet Code** |
| --- | --- | --- | --- | --- | --- | --- | --- | --- | --- | --- | --- |
| 00524115  00527319  01918435 | Benzylpenicillin | Water Soluble Powder | Calves  (**100 kg**) | As an aid in the treatment of **bacterial enteritis**. Administer 4 g of powder per 45 kg of body weight; mix with water or milk and give every 12 hours for 2 or 3 days. | 4,961,333 IU of benzylpenicillin potassium per 100 g of powder, or 4,961,333*0.0006/100 ≈ 29.8 mg of benzylpenicillin per gram of powder | The daily dosage is 4*29.8*2/45 ≈ **5.3** mg/kg/day. The course dosage is 4*29.8*2*2.5/45 ≈ **13.2** mg/kg/course. | **5.3** | **13.2** | 5.3*100 = **530** | 13.2*100 = **1,320** | **QA07AA54** (Intestinal antiinfectives – streptomycin, combinations) |
|  | Streptomycin Sulfate |  |  |  | 153.33 mg of streptomycin sulfate per gram of powder | The daily dosage is 4*153.33*2/45 ≈ **27.3** mg/kg/day. The course dosage is 4*153.33*2*2.5/45 ≈ **68.1** mg/kg/course. | **27.3** | **68.1** | 27.3*100 = **2,730** | 68.1*50 = **6,810** |  |
| 00568120 | Neomycin Sulfate | Water Soluble Powder | Calves  (**100 kg**) | **1-** As an aid in the prevention or treatment of **bacterial enteritis**. Individual treatment: Mix 1 rounded teaspoonful per 100 kg body weight in 100 mL of water for 4 to 5 days.  **2-** As an aid in the prevention or treatment of **bacterial respiratory infections**. Individual treatment: Mix 1 rounded teaspoonful per 50 kg body weight in 100 mL of water or milk for 4 to 5 days.  Approximation used for calculations: A rounded teaspoonful contains 4 grams of powder. | 200 mg of neomycin sulfate per gram of powder | The daily dosage is either 4*200/100 = 8 or 4*200/50 = 16 mg/kg/day, for an average daily dosage of **12** mg/kg/day. The course dosage is either 4.5*8 = 36 or 4.5*16 = 72 mg/kg/day, for an average course dosage of **54** mg/kg/course. | **12** | **54** | 12*100 = **1,200** | 54*100 = **5,400** | **QJ01RA97** (Antibacterials for systemic use – aminoglycosides, combinations with other antibacterials) |
|  | Oxytetracycline |  |  |  | 200 mg of oxytetracycline hydrochloride per gram of powder | The daily dosage is either 4*200/100 = 8 or 4*200/50 = 16 mg/kg/day, for an average daily dosage of **12** mg/kg/day. The course dosage is either 4.5*8 = 36 or 4.5*16 = 72 mg/kg/day, for an average course dosage of **54** mg/kg/course. | **12** | **54** | 12*100 = **1,200** | 54*100 = **5,400** |  |
| 02438313 | Neomycin Sulfate | Water Soluble Powder | Calves  (**100 kg**) | As an aid in the treatment of **bacterial enteritis** and **respiratory diseases**. Individual treatment (scours): dissolve 2 g of powder in 60 mL of water or milk substitute per 45 kg of body weight and give as a drench for 4 to 5 days. Individual treatment (respiratory diseases): double the above dosage for 4 to 5 days. | 200 mg of neomycin sulfate per gram of powder | The daily dosage is 3*200/45 ≈ 13.3 mg/kg/day. The course dosage is 4.5*3*200/45 = 60 mg/kg/course. | **13.3** | **60** | 13.3*100 = **1,330** | 60*100 = **6,000** | **QJ01RA97** (Antibacterials for systemic use – aminoglycosides, combinations with other antibacterials) |
|  | Oxytetracycline |  |  |  | 200 mg of oxytetracycline hydrochloride per gram of powder | The daily dosage is 3*200/45 ≈ 13.3 mg/kg/day. The course dosage is 4.5*3*200/45 = 60 mg/kg/course. | **13.3** | **60** | 13.3*100 = **1,330** | 60*100 = **6,000** |  |
| 00274895  00308242 | Neomycin Sulfate | Oral suspensions | Calves  (**100 kg**) | For use in the treatment of **diarrhea** (scours) and **enteritis**. Give 2 tablespoons orally daily. Treatment may be repeated for 2 to 3 days.  Approximation used for calculations: A tablespoon contains 15 mL. The average duration of treatment is 2.5 days. | 28.6 mg of neomycin sulfate per mL | The daily dosage is 2*15*28.6/50 = **17.16** mg/kg/day. The course dosage is 17.16*2.5 = **42.9** mg/kg/course. | **17.2** | **42.9** | 17.2*100 = **1,720** | 42.9*100 = **4,290** | **QA07AA51** (Intestinal antiinfectives – neomycin, combinations) |
|  | Succinylsulfathiazole |  |  |  | 96.0 mg of succinylsulfathiazole per mL | The daily dosage is 2*15*96/50 = **57.6** mg/kg/day. The course dosage is 57.6*2.5 = **144** mg/kg/course. | **57.6** | **144** | 57.6*100 = **5,760** | 144*100 = **14,400** |  |
| 00641618  02243017 | Neomycin Sulfate | Boluses | Calves  (**100 kg**) | As an aid in the treatment of **bacterial enteritis**, **bacterial respiratory tract infections** and **coccidiosis**. Administer orally 2 bolus per 50 kg of body weight twice daily the first day, and 1 bolus per 50 kg of body weight twice daily for the next 2 to 3 days.  Interpretation: The average dose is 4 bolus per day (day 1) and 2 bolus per day (2.5 additional days) per 50 kg. | 357 mg of neomycin sulfate (250 mg of neomycin base) per bolus | The course dosage is (4+2.5*2)*357/50 = **64.26** mg/kg/course. The daily dosage is 64.26/3.5 = **18.36** mg/kg/day. | **18.4** | **64.3** | 18.4*100 = **1,840** | 64.3*100 = **6,430** | **QJ01RA97** (Antibacterials for systemic use – aminoglycosides, combinations with other antibacterials) |
|  | Sulfamethazine |  |  |  | 2000 mg of sulfamethazine per bolus | The course dosage is (4+2.5*2)*2000/50 = **360** mg/kg/course. The daily dosage is 360/3.5 = **102.9** mg/kg/day. | **102.9** | **360** | 102.9*100 = **10,290** | 360*100 = **36,000** |  |
| 00527335  01976338 | Neomycin Sulfate | Water Soluble Powder | Calves  (**100 kg**) | **1-** As an aid in the treatment of **bacterial enteritis**. Individual treatment: Dissolve 2 g of powder in 60 mL of water or milk substitute per 45 kg of body weight and administer as a drench for 4 or 5 days.  **2-** As an aid in the treatment of **respiratory diseases**. Double the above dosage for 4 to 5 days. | 200 mg of neomycin sulfate per gram of powder | The average daily dosage is 3*200/45 ≈ **13.3** mg/kg/day. The average course dosage is 3*200*4.5/45 = **60** mg/kg/course. | **13.3** | **60** | 13.3*100 = **1,330** | 60*100 = **6,000** | **QJ01RA97** (Antibacterials for systemic use – aminoglycosides, combinations with other antibacterials) |
|  | Tetracycline |  |  |  | 200 mg of tetracycline hydrochloride per gram of powder | The average daily dosage is 3*200/45 ≈ **13.3** mg/kg/day. The average course dosage is 3*200*4.5/45 = **60** mg/kg/course. | **13.3** | **60** | 13.3*100 = **1,330** | 60*100 = **6,000** |  |
| 00260061 | Sulfamethazine | Water Soluble Powder | All cattle including lactating dairy animals  (**300 kg**) | As an aid in the prevention and treatment of bacterial respiratory infections and bacterial enteritis. Cattle: shipping fever, enteritis, foot rot, and winter dysentery.  Treatment level: 30 g of powder per 23 liters, or 454.4 g per 380 liters, or 3.6 kg per 760 liters. Make only sufficient solution for 24 hours.  Calves: One tablespoon per 45.4 kg of bodyweight for treatment. Use in water, milk, or milk replacer. Continue medication for 7 to 10 days.  Approximation used for calculations: A tablespoon contains 9 grams of powder. | 20 g of sulfamethazine sodium per 454.4 g of powder  (44.0 mg per g of powder) | Using the individual treatment dosage (for calves): The daily dosage is 9*44.0/45.4 ≈ **8.7** mg/kg/day. The course dosage is 8.5*9*44.0/45.4 ≈ **74.1** mg/kg/course. | **8.7** | **74.1** | 8.7*300 = **2,610** | 74.1*300 = **22,230** | **QJ01EQ30** (Antibacterials for systemic use – combination of sulfonamides) |
|  | Sulfathiazole |  |  |  | 180 g of sulfathiazole sodium per 454.4 g of powder  (396.1 mg per g of powder) | Using the individual treatment dosage (for calves): The daily dosage is 9*396.1/45.4 ≈ **78.5** mg/kg/day. The course dosage is 8.5*9*396.1/45.4 ≈ **667.4** mg/kg/course. | **78.5** | **667.4** | 78.5*300 = **23,550** | 667.4*300 = **200,220** |  |
| 02030586 | Sulfamethazine | Water Soluble Powder | Cattle other than lactating dairy animals  (**300 kg**) | As an aid in the treatment of infections such as pneumonia, metritis, enteritis, foot rot, mastitis and complicated viral infections (BRD Complex). Individual treatment: On the first day, 60 mL of a 12.5% solution or 30 mL of a 25% solution per 35 kg of body weight. On the second, third and fourth day, administer half of the dosage of the first day. For a 12.5% solution, dissolve 567 g in water to make a total volume of 4.5 litres of solution. For a 25% solution, dissolve 567 g in water to make a total volume of 2.27 litres of solution. | 630 mg of sulfamethazine sodium per gram of powder (79.38 mg per mL of the 12.5% solution, or 157.36 mg per mL of the 25% solution) | The course dosage is either (60*79.38+30*79.38*3)/35 = **340.2** mg/kg/course or (30*157.36+15*157.36*3)/35 = **337.2** mg/kg/course.  The average course dosage is (340.2+337.2)/2 = **338.7** mg/kg/course. The daily dosage is 338.7/4 ≈ **84.7** mg/kg/day. | **84.7** | **338.7** | 84.7*300 = **25,410** | 338.7*300 = **101,610** | **QJ01EQ30** (Antibacterials for systemic use – combination of sulfonamides) |
|  | Sulfathiazole |  |  |  | 315 mg of sulfathiazole sodium per gram of powder (39.69 mg per mL of the 12.5% solution, or 78.68 mg per mL of the 25% solution) | The course dosage is either (60*39.69+30*39.69*3)/35 = **170.1** mg/kg/course or (30*78.68+15*78.68*3)/35 = **168.6** mg/kg/course.  The average course dosage is (170.1+168.6)/2 = **169.35** mg/kg/course. The daily dosage is 169.35/4 ≈ **42.3** mg/kg/day. | **42.3** | **169.4** | 42.3*300 = **12,690** | 169.4*300 = **50,820** |  |
| 00337552  00644803 | Sulfamethazine | Water Soluble Powder | All cattle including lactating dairy animals  (**300 kg**) | For the treatment of bacterial infections (pneumonia, metritis, enteritis, foot rot, or septicemia accompanying mastitis) and as an aid in the treatment of shipping fever complex. For individual treatment, dissolve 300 grams in 1.5 litres of water (or 400 grams in 2 litres) and administer 1 mL of this solution per kg bodyweight as a drench. Reduce the dose to 0.5 mL/kg bodyweight for the next 3 days. | 667 mg of sulfamethazine sodium per gram of powder (133.4 mg per mL of the solution) | The course dosage is 133.4+0.5*133.4*3 = **333.5** mg/kg/course. The daily dosage is 333.5/4 = **83.375** mg/kg/day. | **83.4** | **333.5** | 83.4*300 = **25,020** | 333.5*300 = **100,050** | **QJ01EQ30** (Antibacterials for systemic use – combination of sulfonamides) |
|  | Sulfathiazole |  |  |  | 333 mg of sulfathiazole sodium per gram of powder (66.6 mg per mL of the solution) | The course dosage is 66.6+0.5*66.6*3 = **166.5** mg/kg/course. The daily dosage is 166.5/4 = **41.625** mg/kg/day. | **41.6** | **166.5** | 41.6*300 = **12,480** | 166.5*300 = **49,950** |  |
| 02229489 | Sulfamethazine | Water Soluble Powder | All cattle including lactating dairy animals  (**300 kg**) | As an aid in the treatment of infections such as pneumonia, metritis, enteritis, foot rot and mastitis. For individual treatment: Dissolve 400 g in 2 L of water and administer 1 mL of this solution per kg of body weight as a drench. Reduce the dose to 0.5 mL per kg of body weight for the next 3 days. | 630 mg of sulfamethazine sodium per gram of powder (126 mg per mL of the solution) | The course dosage is 126+0.5*126*3 = **315** mg/kg/course. The daily dosage is 315/4 = **78.75** mg/kg/day. | **78.8** | **315** | 78.8*300 = **23,640** | 315*300 = **94,500** | **QJ01EQ30** (Antibacterials for systemic use – combination of sulfonamides) |
|  | Sulfathiazole |  |  |  | 315 mg of sulfathiazole sodium per gram of powder (63 mg per mL of the solution) | The course dosage is 63+0.5*63*3 = **157.5** mg/kg/course. The daily dosage is 157.5/4 ≈ **39.4** mg/kg/day. | **39.4** | **157.5** | 39.4*300 = **11,820** | 157.5*300 = **47,250** |  |

**Supplementary 4.2. Details of the 9 oral products other than medicated premixes, containing exactly three antimicrobial agents, and marketed in Canada with at least one indication for cattle.**

Legend: **ATCvet**: Anatomical Therapeutic Chemical Classification System for veterinary medicinal products; **BRD**: Bovine Respiratory Disease; **DIN**: Drug Identification Number.

| **DIN of products marketed in Canada** | **Antimicrobial agent** | **Formulation** | **Type of cattle targeted by the product** | **Product Monograph** | **Concentration in the product** | **Calculations from the product monograph** | **Daily dosage (mg/kg/day)** | **Course dosage (mg/kg/course)** | **Daily dose (mg/animal/day)** | **Course dose (mg/animal/course)** | **ATCvet Code** |
| --- | --- | --- | --- | --- | --- | --- | --- | --- | --- | --- | --- |
| 00260045  00308390 | Neomycin Sulfate | Boluses | Calves  (**100 kg**) | As an aid in the treatment and prevention of **diarrhea** (scours) and **enteritis** of bacterial origin. 1 bolus per 45 kg body weight orally once daily for 3-4 days if necessary.  Interpretation: The average duration of treatment is 3.5 days. | 400 mg of neomycin sulfate (350 mg of neomycin base) per bolus | The daily dosage is 400/45 ≈ **8.9** mg/kg/day. The course dosage is 400*3.5/45 ≈ **31.1** mg/kg/course. | **8.9** | **31.1** | 8.9*100 = **890** | 31.1*100 = **3,110** | **QA07AA51** (Intestinal antiinfectives – neomycin, combinations) |
|  | Sulfaguanidine |  |  |  | 1340 mg of sulfaguanidine per bolus | The daily dosage is 1340/45 ≈ **29.8** mg/kg/day. The course dosage is 1340*3.5/45 ≈ **104.2** mg/kg/course. | **29.8** | **104.2** | 29.8*100 = **2,980** | 104.2*100 = **10,420** |  |
|  | Sulfathiazole |  |  |  | 668 mg of sulfathiazole per bolus | The daily dosage is 668/45 ≈ **14.8** mg/kg/day. The course dosage is 668*3.5/45 ≈ **52.0** mg/kg/course. | **14.8** | **52.0** | 14.8*100 = **1,480** | 52.0*100 = **5,200** |  |
| 00260126  00308404 | Sulfamethazine | Boluses | All cattle including lactating dairy animals  (**300 kg**) | As an aid in treatment of bacterial pneumonia, metritis, foot rot, diphtheria, and other conditions in cattle when caused by sulfonamide susceptible organisms. Initial dose: 1 bolus per 80 kg bodyweight. Subsequent dose: ½ the initial dose and may be given 12 hours later if necessary. The initial dose is usually sufficient. If no improvement is noted within 4 days, the diagnosis should be re-evaluated.  Interpretation: The course dose is either 1 bolus or 1.5 bolus, for an average of 1.25 bolus per 80 kg of body weight. The duration of action is supposed to be 24h. | 3.90 g of sulfamethazine per bolus | The average course dosage is 1.25*3900/80 ≈ **60.9** mg/kg/course. The average daily dose is **60.9** mg/kg/day. | **60.9** | **60.9** | 60.9*300 = **18,270** | 60.9*300 = **18,270** | **QJ01EQ30** (Antibacterials for systemic use – combination of sulfonamides) |
|  | Sulfanilamide |  |  |  | 5.85 g of sulfanilamide per bolus | The average course dosage is 1.25*5850/80 ≈ **91.4** mg/kg/course. The average daily dose is **91.4** mg/kg/day. | **91.4** | **91.4** | 91.4*300 = **27,420** | 91.4*300 = **27,420** |  |
|  | Sulfathiazole |  |  |  | 5.85 g of sulfathiazole per bolus | The average course dosage is 1.25*5850/80 ≈ **91.4** mg/kg/course. The average daily dose is **91.4** mg/kg/day. | **91.4** | **91.4** | 91.4*300 = **27,420** | 91.4*300 = **27,420** |  |
| 00346632  02240309 | Sulfamethazine | Oral solutions | All cattle including lactating dairy animals  (**300 kg**) | For the treatment of infections for which the causative organisms are susceptible to sulfonamides: foot rot, shipping fever complex, septicemia, bacterial enteritis, upper respiratory infections of bacterial origin, acute mastitis, calf diphtheria, omphalitis, and secondary infections associated with bacterial scours. Initial dose is 75 mL per 45.4 kg of body weight for the first day. Subsequent dose is 25 mL per 45.4 kg of body weight every twelve hours for 2 to 4 additional days.  Interpretation: 75 mL (day 1) and 50 mL (3 additional days) per 45.4 kg of body weight. | 45 mg of sulfamethazine per mL | The average course dosage is (75*45+50*45*3)/45.4 ≈ **223.0** mg/kg/course. The average daily dosage is 223.0/4 ≈ **55.8** mg/kg/day. | **55.8** | **223.0** | 55.8*300 = **16,740** | 223.0*300 = **66,900** | **QJ01EQ30** (Antibacterials for systemic use – combination of sulfonamides) |
|  | Sulfapyridine |  |  |  | 20 mg of sulfapyridine per mL | The average course dosage is (75*20+50*20*3)/45.4 ≈ **99.1** mg/kg/course. The average daily dosage is 99.1/4 ≈ **24.8** mg/kg/day. | **24.8** | **99.1** | 24.8*300 = **7,440** | 99.1*300 = **29,730** |  |
|  | Sulfathiazole |  |  |  | 45 mg of sulfathiazole per mL | The average course dosage is (75*45+50*45*3)/45.4 ≈ **223.0** mg/kg/course. The average daily dosage is 223.0/4 ≈ **55.8** mg/kg/day. | **55.8** | **223.0** | 55.8*300 = **16,740** | 223.0*300 = **66,900** |  |
| 00721069 | Sulfamerazine | Water Soluble Powder | Cattle other than lactating dairy animals  (**300 kg**) | As an aid in the treatment of respiratory and enteric infections.  No individual dosage. Dissolve 400 g in 1500 L of drinking water for 4 to 5 days.  Approximation used for calculations: A daily water intake of 10% is used. | 60 g of sodium sulfamerazine per 400 g of powder | The daily dosage is (60/1500)*1000*10% = **4.0** mg/kg/day. The course dosage is 4.0*4.5 = **18** mg/kg/course. | **4.0** | **18** | 4*300 = **1,200** | 18*300 = **5,400** | **QJ01EQ30** (Antibacterials for systemic use – combination of sulfonamides) |
|  | Sulfamethazine |  |  |  | 150 g of sodium sulfamethazine per 400 g of powder | The daily dosage is (150/1500)*1000*10% = **10** mg/kg/day. The course dosage is 10*4.5 = **45** mg/kg/course. | **10** | **45** | 10*300 = **3,000** | 45*300 = **13,500** |  |
|  | Sulfathiazole |  |  |  | 120 g of sodium sulfathiazole per 400 g of powder | The daily dosage is (120/1500)*1000*10% = **8** mg/kg/day. The course dosage is 8*4.5 = **36** mg/kg/course. | **8** | **36** | 8*300 = **2,400** | 36*300 = **10,800** |  |
| 01985299 | Sulfamerazine | Water Soluble Powder | Cattle other than lactating dairy animals  (**300 kg**) | As an aid in the prevention and treatment of infections such as bacterial enteritis, bacterial pneumonia and salmonellosis.  No individual dosage. Dissolve 450 g in 1350 L of drinking water for 5 to 10 days.  Approximation used for calculations: A daily water intake of 10% is used. | 67.4 g of sulfamerazine sodium per 450 g of powder | The daily dosage is (67.4/1350)*1000*10% ≈ **5.0** mg/kg/day. The course dosage is 5.0*7.5 ≈ **37.4** mg/kg/course. | **5.0** | **37.4** | 5*300 = **1,500** | 37.4*300 = **11,220** | **QJ01EQ30** (Antibacterials for systemic use – combination of sulfonamides) |
|  | Sulfamethazine |  |  |  | 170.5 g of sulfamethazine sodium per 450 g of powder | The daily dosage is (170.5/1350)*1000*10% ≈ **12.6** mg/kg/day. The course dosage is 12.6*7.5 ≈ **94.7** mg/kg/course. | **12.6** | **94.7** | 12.6*300 = **3,780** | 94.7*300 = **28,410** |  |
|  | Sulfathiazole |  |  |  | 136.3 g of sulfathiazole sodium per 450 g of powder | The daily dosage is (136.3/1350)*1000*10% ≈ **10.1** mg/kg/day. The course dosage is 10.1*7.5 ≈ **75.7** mg/kg/course. | **10.1** | **75.7** | 10.1*300 = **3,030** | 75.7*300 = **22,710** |  |
| 00511757 | Sulfamerazine | Water Soluble Powder | Cattle other than lactating dairy animals  (**300 kg**) | As an aid in the prevention and treatment of infections such as bacterial enteritis, bacterial pneumonia, and salmonellosis. It can also be used as an aid in the treatment of complicated viral infections in cattle (BRD complex or shipping fever).  No individual dosage. Dissolve 450 g in 1350 L of drinking water for 5 to 10 days.  Approximation used for calculations: A daily water intake of 10% is used. | 67.4 g of sulfamerazine sodium per 450 g of powder | The daily dosage is (67.4/1350)*1000*10% ≈ **5.0** mg/kg/day. The course dosage is 5.0*7.5 ≈ **37.4** mg/kg/course. | **5.0** | **37.4** | 5*300 = **1,500** | 37.4*300 = **11,220** | **QJ01EQ30** (Antibacterials for systemic use – combination of sulfonamides) |
|  | Sulfamethazine |  |  |  | 170.5 g of sulfamethazine sodium per 450 g of powder | The daily dosage is (170.5/1350)*1000*10% ≈ **12.6** mg/kg/day. The course dosage is 12.6*7.5 ≈ **94.7** mg/kg/course. | **12.6** | **94.7** | 12.6*300 = **3,780** | 94.7*300 = **28,410** |  |
|  | Sulfathiazole |  |  |  | 102.9 g of sulfathiazole sodium per 450 g of powder | The daily dosage is (102.9/1350)*1000*10% ≈ **7.6** mg/kg/day. The course dosage is 7.6*7.5 ≈ **57.2** mg/kg/course. | **7.6** | **57.2** | 7.6*300 = **2,280** | 57.2*300 = **17,160** |  |

**Supplementary 4.3. Average daily dosage, course dosage, daily dose, and course dose, for neomycin sulfate, oxytetracycline, sulfamerazine, sulfamethazine (sulfadimidine), and sulfathiazole (from oral products other than medicated premixes, containing more than one antimicrobial agent).**

1. **Neomycin Sulfate**

Combination with oxytetracycline (water soluble powders): The daily dosage is (12+13.3)/2 ≈ **12.7 mg/kg/day**. The course dosage is (54+60)/2 = **57 mg/kg/course**. The daily dose is (1.2+1.33)/2 ≈ **1.27 g/animal/day**. The course dose is (5.4+6.0)/2 = **5.7 g/animal/course**.

Combination with succinylsulfathiazole (oral suspensions): The daily dosage is **17.2 mg/kg/day**. The course dosage is **42.9 mg/kg/course**. The daily dose is **1.72 g/animal/day**. The course dose is **4.29 g/animal/course**.

Combination with sulfamethazine (boluses): The daily dosage is **18.4 mg/kg/day**. The course dosage is **64.3 mg/kg/course**. The daily dose is **1.84 g/animal/day**. The course dose is **6.43 g/animal/course**.

Combination with tetracycline (water soluble powders): The daily dosage is **13.3 mg/kg/day**. The course dosage is **60 mg/kg/course**. The daily dose is **1.33 g/animal/day**. The course dose is **6.0 g/animal/course**.

Combination with sulfaguanidine and sulfathiazole (boluses): The daily dosage is **8.9 mg/kg/day**. The course dosage is **31.1 mg/kg/course**. The daily dose is **0.89 g/animal/day**. The course dose is **3.11 g/animal/course**.

Average values (boluses, suspensions, water soluble powders): The average daily dosage is (12.7+17.2+18.4+13.3+8.9)/5 = **14.1 mg/kg/day**. The average course dosage is (57+42.9+64.3+60+31.1)/5 ≈ **51.1 mg/kg/course**. The average daily dose is (1.27+1.72+1.84+1.33+0.89)/5 = **1.41 g/animal/day**. The average course dose is (5.7+4.29+6.43+6.0+3.11)/5 ≈ **5.11 g/animal/course**.

1. **Oxytetracycline**

Combination with neomycin (water soluble powders): The daily dosage is (12+13.3)/2 ≈ **12.7 mg/kg/day**. The course dosage is (54+60)/2 = **57 mg/kg/course**. The daily dose is (1.2+1.33)/2 ≈ **1.27 g/animal/day**. The course dose is (5.4+6.0)/2 = **5.7 g/animal/course**.

1. **Sulfamerazine**

Combination with sulfamethazine and sulfathiazole (water soluble powders): The daily dosage is (4+5)/2 = **4.5 mg/kg/day**. The course dosage is (18+37.4)/2 = **27.7 mg/kg/course**. The daily dose is (1.2+1.5)/2 = **1.35 g/animal/day**. The course dose is (5.4+11.22)/2 = **8.31 g/animal/course**.

1. **Sulfamethazine (Sulfadimidine)**

Combination with neomycin (boluses): The daily dosage is **102.9 mg/kg/day**. The course dosage is **360 mg/kg/course**. The daily dose is **10.29 g/animal/day**. The course dose is **36.0 g/animal/course**.

Combination with sulfathiazole (water soluble powders): The daily dosage is (8.7+84.7+83.4+78.8)/4 = **63.9 mg/kg/day**. The course dosage is (74.1+338.7+333.5+315)/4 ≈ **265.3 mg/kg/course**. The daily dose is (2.61+25.41+25.02+23.64)/4 = **19.17 g/animal/day**. The course dose is (22.23+101.61+100.05+94.5)/4 = **79.60 g/animal/course**.

Combination with sulfanilamide and sulfathiazole (boluses): The daily dosage is **60.9 mg/kg/day**. The course dosage is **60.9 mg/kg/course**. The daily dose is **18.27 g/animal/day**. The course dose is **18.27 g/animal/course**.

Combination with sulfapyridine and sulfathiazole (oral solutions): The daily dosage is **55.8 mg/kg/day**. The course dosage is **223 mg/kg/course**. The daily dose is **16.74 g/animal/day**. The course dose is **66.9 g/animal/course**.

Combination with sulfamerazine and sulfathiazole (water soluble powders): The daily dosage is (10+12.6)/2 = **11.3 mg/kg/day**. The course dosage is (45+94.7)/2 ≈ **69.9 mg/kg/course**. The daily dose is (3+3.78)/2 = **3.39 g/animal/day**. The course dose is (13.5+28.41)/2 ≈ **20.96 g/animal/course**.

Average values (boluses, solutions, water soluble powders): The average daily dosage is (102.9+63.9+60.9+55.8+11.3)/5 ≈ **59.0 mg/kg/day**. The average course dosage is (360+265.3+60.9+223+69.9)/5 ≈ **195.8 mg/kg/course**. The average daily dose is (10.29+19.17+18.27+16.74+3.39)/5 ≈ **13.57 g/animal/day**. The average course dose is (36.0+79.6+18.27+66.9+20.96)/5 ≈ **44.35 g/animal/course**.

1. **Sulfathiazole**

Combination with sulfamethazine (water soluble powders): The daily dosage is (78.5+42.3+41.6+39.4)/4 ≈ **50.5 mg/kg/day**. The course dosage is (667.4+169.4+166.5+157.5)/4 = **290.2 mg/kg/course**. The daily dose is (23.55+12.69+12.48+11.82)/4 ≈ **15.14 g/animal/day**. The course dose is (200.22+50.82+49.95+47.25)/4 = **87.06 g/animal/course**.

Combination with neomycin and sulfaguanidine (boluses): The daily dosage is **14.8 mg/kg/day**. The course dosage is **52.0 mg/kg/course**. The daily dose is **1.48 g/animal/day**. The course dose is **5.2 g/animal/course**.

Combination with sulfamethazine and sulfanilamide (boluses): The daily dosage is **91.4 mg/kg/day**. The course dosage is **91.4 mg/kg/course**. The daily dose is **27.42 g/animal/day**. The course dose is **27.42 g/animal/course**.

Combination with sulfamethazine and sulfapyridine (oral solutions): The daily dosage is **55.8 mg/kg/day**. The course dosage is **223.0 mg/kg/course**. The daily dose is **16.74 g/animal/day**. The course dose is **66.9 g/animal/course**.

Combination with sulfamerazine and sulfamethazine (water soluble powders): The daily dosage is (8+10.1+7.6)/3 ≈ **8.6 mg/kg/day**. The course dosage is (36+75.7+57.2)/3 = **56.3 mg/kg/course**. The daily dose is (2.4+3.03+2.28)/3 = **2.57 g/animal/day**. The course dose is (10.8+22.71+17.16)/3 = **16.89 g/animal/course**.

Average values (boluses, solutions, water soluble powders): The average daily dosage is (50.5+14.8+91.4+55.8+8.6)/5 ≈ **44.2 mg/kg/day**. The average course dosage is (290.2+52.0+91.4+223.0+56.3)/5 ≈ **142.6 mg/kg/course**. The average daily dose is (15.14+1.48+27.42+16.74+2.57)/5 ≈ **12.67 g/animal/day**. The average course dose is (87.06+5.2+27.42+66.9+16.89)/5 ≈ **40.69 g/animal/course**.

**Supplementary 5.1. Details of the 8 intramammary products containing at least one antimicrobial agent, and marketed for cattle in Canada.**

Legend: **ATCvet**: Anatomical Therapeutic Chemical Classification System for veterinary medicinal products; **DIN**: Drug Identification Number; **IU**: International Unit(s).

Arbitrary decisions: “For IMM products for lactating cows, it was hypothesized that one quarter is infected (and treated) at a time by cow. For IMM products for dry cows, 2 arbitrary decisions were applied. First decision: a complete treatment for a cow was defined as the infusion of four syringes (one per quarter) at drying off regardless of the product. Second decision: the complete treatment (4 syringes) was assigned to a duration of action of 10 days, meaning that the daily treatment was defined as the infusion of 0.4 syringe per cow, or 0.1 syringe per quarter.”

| **DIN of products marketed in Canada** | **Antimicrobial agent(s)** | **Mg of AM per 10-mL disposable syringe** | **Type of cattle targeted by the product** | **Indication(s)** | **Product Monograph** | **Daily dose (mg/animal/day)** | **Course dose (mg/animal/course)** | **Daily dose (unit dose/animal/day)** | **Course dose (unit dose/animal/course)** | **ATCvet Code** |
| --- | --- | --- | --- | --- | --- | --- | --- | --- | --- | --- |
| 02173263 | Cefapirin | 200 | Lactating cows | For the **treatment of mastitis** caused by susceptible strains of *Streptococcus agalactiae* and *Staphylococcus aureus* including strains resistant to penicillin. | Infuse the entire contents of one syringe into each infected quarter immediately after the quarter has been completely milked out. Repeat once only in 12 hours. | **400** | **400** | **2** | **2** | **QJ51DB08** (Other beta-lactam antibacterials for IMM use – first-generation cephalosporins) |
| 02298325 | Ceftiofur | 125 | Lactating cows | For (1) the **treatment of clinical mastitis** caused by *Escherichia coli*, *Streptococcus dysgalactiae* and coagulase negative staphylococci and (2) the **treatment of diagnosed subclinical mastitis** associated with coagulase-negative staphylococci and *Streptococcus dysgalactiae*. | Infuse one syringe into each infected quarter. Repeat this treatment once in 24 hours. | **125** | **250** | **1** | **2** | **QJ51DD90** (Other beta-lactam antibacterials for IMM use – third-generation cephalosporins) |
| 02264730 | Pirlimycin | 50 | Lactating cows | For the **treatment of clinical and subclinical mastitis** caused by *Staphylococcus aureus*, and **treatment of subclinical mastitis** caused by *Streptococcus dysgalactiae* and *Streptococcus uberis*. | Dosage1: Infuse one syringe into each infected quarter. Repeat this treatment once after a 24-hour interval.  Dosage2: Infuse each infected quarter daily for 8 consecutive days (extended duration therapy). | **50** | (2*50+8*50)/2 = **250** | **1** | (2+8)/2 = **5** | **QJ51FF90** (Macrolides and lincosamides for IMM use – lincosamides) |
| 00813877 | Benzylpenicillin Procaine | 60  (100,000 IU) | Lactating cows | For the **treatment of acute and chronic bovine mastitis** caused by susceptible strains of staphylococci, streptococci, coliforms or pseudomonads. | Infuse one syringe per quarter. Repeat once after a 24-hour period if necessary.  **Communication with the pharmaceutical company: only the infected quarter has to be treated.**  **Interpretation: A complete treatment includes one syringe or 2 syringes (average 1.5 syringes).** | **60** | 60*1.5 = **90** | **1** | **1.5** | **QJ51RV01** (Combinations of antibacterials and other substances for IMM use – antibacterials and corticosteroids) |
|  | Dihydrostreptomycin | 100 |  |  |  | **100** | 100*1.5 = **150** | **1** | **1.5** |  |
|  | Novobiocin | 150 |  |  |  | **150** | 150*1.5 = **225** | **1** | **1.5** |  |
|  | Polymyxin B Sulfate | 6  (50,000 IU) |  |  |  | **6** | 6*1.5 = **9** | **1** | **1.5** |  |
| 02173255 | Cefapirin | 300 | Dry cows | For the **treatment of mastitis** caused by *Streptococcus agalactiae* in dairy cows during the dry period. Treatment is indicated in any cow known to harbour any of these organisms in the udder at drying off. | Infuse each quarter at the time of drying off with a single 10 mL syringe. | **120** | **1,200** | **0.4** | **4** | **QJ51DB08** (Other beta-lactam antibacterials for IMM use – first-generation cephalosporins) |
| 02312654 | Ceftiofur | 500 | Dry cows | For the **treatment of subclinical mastitis** associated with *Staphylococcus aureus*, *Streptococcus dysgalactiae* and *Streptococcus uberis* at the time of dry-off. | Infuse one syringe into each affected quarter at the time of dry-off.  **Arbitrary decision: 4 syringes per quarter at the time of drying off.** | **200** | **2000** | **0.4** | **4** | **QJ51DD90** (Other beta-lactam antibacterials for IMM use – third-generation cephalosporins) |
| 02173247 | Cloxacillin | 500 | Dry cows | For the **treatment of mastitis** caused by *Streptococcus agalactiae* and *Staphylococcus aureus* in dairy cows during the dry period. Treatment is indicated in any cow known to harbour any of these organisms in the udder at drying off, or which has had repeated attacks of mastitis during the previous lactation, or is affected with mastitis at drying off, if caused by susceptible organisms. | Infuse the contents of one syringe (10 mL) into each quarter immediately following the last milking or early in the dry period. | **200** | **2,000** | **0.4** | **4** | **QJ51CF02** (Beta-lactamase resistant penicillins for IMM use) |
| 00813850 | Benzylpenicillin Procaine | 120  (200,000 IU) | Dry cows | For the **treatment of mastitis** caused by *Staphylococcus aureus* and/or *Streptococcus agalactiae*, and for the **prevention of mastitis** caused by *Streptococcus agalactiae*. | Dosage: 10 mL per quarter immediately after drying off. | **48** | **480** | **0.4** | **4** | **QJ51RC23** (Combinations of antibacterials for IMM use – procaine benzylpenicillin, combinations with other antibacterials) |
|  | Novobiocin | 400 |  |  |  | **160** | **1,600** | **0.4** | **4** |  |

**Supplementary 5.2. Details of the 5 intrauterine products containing at least one antimicrobial agent, and marketed in Canada with at least one indication for cattle.**

Legend: **ATCvet**: Anatomical Therapeutic Chemical Classification System for veterinary medicinal products; **DIN**: Drug Identification Number.

Arbitrary decision: “For intrauterine products, when no indication could be found on the label, a duration of action of 24 hours was assigned.”

| **DIN of products marketed in Canada** | **Antimicrobial agent(s)** | **Dosage form** | **Mg of AM per unit dose** | **Type of cattle targeted by the product** | **Product Monograph** | **Daily dose (mg/animal/day)** | **Course dose (mg/animal/course)** | **ATCvet Code** |
| --- | --- | --- | --- | --- | --- | --- | --- | --- |
| 02237680 | Cefapirin | Suspension in a disposable single-use syringe | 500 mg of cefapirin per syringe (640 mg of cefapirin benzathine) | Beef and dairy cows | Introduce a single dose of 640 mg of cephapirin benzathine into the lumen of the uterus using a disposable catheter. One treatment is usually sufficient for a complete cure. If the quantity and quality of the cervical discharge is not improved at examination 14 days after treatment, the cow should receive a second treatment. After a single treatment, concentrations of cephapirin in endometrial tissue above the MIC of sensitive bacteria are maintained for at least 24 hours.  Interpretation: The course dose is 1 syringe per cow. The duration of action based on the label is 1 day. | **500** | **500** | **QG51AA05** (Antiinfectives and antiseptics for intrauterine use – antibacterials) |
| 00592293 | Gentamicin Sulfate | Solution | 100 mg/mL | Beef cows only | Cows: 200 mg one time only. The dose should be diluted with 16 mL sterile physiological saline solution before aseptic uterine infusion.  Interpretation: No information on the duration of action (by default 24 hours). | **200** | **200** | **QG51AA04** (Antiinfectives and antiseptics for intrauterine use – antibacterials) |
| 00439339 | Oxytetracycline Hydrochloride | Suspension | 50 mg/mL | Beef and dairy cows | Antibiotics are infused through a pipette into the uterus for the treatment of metritis.  Weight of animal – mL to administer  600 lb (272.2 kg) - 21 to 24 mL  800 lb (362.9 kg) - 28 to 32 mL  1000 lb (453.6 kg) - 35 to 40 mL  1200 lb (544.3 kg) - 42 to 48 mL  1500 lb (680.4 kg) - 53 to 60 mL  Interpretation: For an adult cow weighing 650 kg, an average of 50 mL is infused. No information on the duration of action (by default 24 hours). | **2,500** | **2,500** | **QG51AA01** (Antiinfectives and antiseptics for intrauterine use – antibacterials) |
| 00237531  00308226 | Sulfanilamide | Boluses | 1920 mg/bolus | Beef and dairy cows | Insert one or two boluses deeply into the uterus.  Interpretation: The course dose is 1.5 boluses. No information on the duration of action (by default 24 hours). | **2,880** | **2,880** | **QG01AE10** (Gynecological antiinfectives and antiseptics, excl. combinations with corticosteroids – combinations of sulfonamides) |
|  | Sulfathiazole |  | 320 mg/bolus |  |  | **480** | **480** |  |

**Supplementary 5.3. Details of the 4 topical products containing at least one antimicrobial agent, and marketed in Canada with at least one indication for cattle.**

Legend: **ATCvet**: Anatomical Therapeutic Chemical Classification System for veterinary medicinal products; **DIN**: Drug Identification Number.

Arbitrary decisions: “For topical products, we assumed that 1 mL is sprayed per second (sprays), 5 grams of cream is used per application (creams), or 5 grams of powder is used per application (powders for topical administration).”

| **DIN of products marketed in Canada** | **Antimicrobial agent(s)** | **Dosage form and concentration** | **Type of cattle targeted by the product** | **Product monograph** | **Calculations from the product monograph** | **Daily dose (mg/animal/day)** | **Course dose (mg/animal/course)** | **ATCvet Code** |
| --- | --- | --- | --- | --- | --- | --- | --- | --- |
| 02462419 | Chlortetracycline Hydrochloride | Spray  2.45% w/w  (24.5 mg/mL) | All cattle | Prevention: As an aid in the prevention of infections of superficial traumatic or surgical wounds caused by microorganisms sensitive to chlortetracycline.  Treatment: As an aid in the treatment of active lesions of digital dermatitis caused by organisms sensitive to chlortetracycline.  Spray for approximately 3 seconds until the treatment-area is evenly coloured. For prevention of infections, a single administration is recommended. As an aid in treatment of digital dermatitis active lesions, a double administration (with a 30 second interval) is recommended daily for 3 consecutive days. | Approximation used for calculations: 1 mL is sprayed per second.  The treatment dose is used for calculations. The daily dose is 2*3*24.5 = **147** mg/animal/day. The course dose is 147*3 = **441** mg/kg/course. | **147** | **441** | **QD06AA02** (Antibiotics and chemotherapeutics for dermatological use – antibiotics for topical use – tetracycline and derivatives) |
| 00502650  00502936 | Sulfanilamide | 38.8 mg per gram of cream | All cattle | A topical antibacterial agent for use in cattle for the treatment of infections caused by bacteria susceptible to sulfonamides. Also for use in the treatment of skin conditions.  Apply an amount sufficient to cover the affected tissue twice daily. | Approximation used for calculations: 5 g of cream per application, twice daily for 5 days.  The daily dose is 5*38.8*2 = **388** mg/animal/day. The course dose is 388*5 = **1940** mg/animal/course. | **388** | **1,940** | **QD06BA99** (Antibiotics and chemotherapeutics for dermatological use – chemotherapeutics for topical use – sulfonamides, combinations) |
|  | Sulfathiazole | 38.8 mg per gram of cream |  |  | Approximation used for calculations: 5 g of cream per application, twice daily for 5 days.  The daily dose is 5*38.8*2 = **388** mg/animal/day. The course dose is 388*5 = **1940** mg/animal/course. | **388** | **1,940** |  |
| 00346152 | Sulfanilamide | Powder containing 5% sulfanilamide  (50 mg/g) | All cattle | A counter-irritant, astringent and antimicrobial powder for use in cattle for slow healing wounds with a tendency towards proud flesh (exuberant granulations).  Dust wounds twice daily. Repeat as indicated. | Approximation used for calculations: 5 g of powder per application, twice daily for 5 days.  The daily dose is 5*50*2 = **500** mg/animal/day. The course dosage is 500*5 = **2500** mg/animal/course. | **500** | **2,500** | **QD06BA99** (Antibiotics and chemotherapeutics for dermatological use – chemotherapeutics for topical use – sulfonamides, combinations) |
|  | Sulfathiazole | Powder containing 5% sulfathiazole  (50 mg/g) |  |  | Approximation used for calculations: 5 g of powder per application, twice daily for 5 days.  The daily dose is 5*50*2 = **500** mg/animal/day. The course dosage is 500*5 = **2500** mg/animal/course. | **500** | **2,500** |  |

Sulfanilamide: The daily dose is (388+500)/2 = **444 mg/animal/day**. The course dose is (1940+2500)/2 = **2,220 mg/animal/course**.

Sulfathiazole: The daily dose is (388+500)/2 = **444 mg/animal/day**. The course dose is (1940+2500)/2 = **2,220 mg/animal/course**.
